# Phylogenetic Analysis and Comparative Genomics of *Brucella abortus* and *Brucella melitensis* Strains in Egypt

**DOI:** 10.1007/s00239-024-10173-0

**Published:** 2024-05-29

**Authors:** Alyaa Elrashedy, Mohamed Nayel, Akram Salama, Ahmed Zaghawa, Nader R. Abdelsalam, Mohamed E. Hasan

**Affiliations:** 1 Department of Animal Medicine and Infectious Diseases (Infectious Diseases), Faculty of Veterinary Medicine, University of Sadat City, Sadat City, Egypt; 2 Agricultural Botany Department, Faculty of Agriculture (Saba Basha), Alexandria University, Alexandria, 21531 Egypt; 3 Bioinformatics Department, Genetic Engineering and Biotechnology Research Institute, University of Sadat City, Sadat City, Egypt

**Keywords:** *Brucella*, Genome annotation, Comparative genome analysis, Phylogenetic analysis, Bioinformatics

## Abstract

Brucellosis is a notifiable disease induced by a facultative intracellular *Brucella* pathogen. In this study, eight *Brucella abortus* and eighteen *Brucella melitensis* strains from Egypt were annotated and compared with RB51 and REV1 vaccines respectively. RAST toolkit in the BV-BRC server was used for annotation, revealing genome length of 3,250,377 bp and 3,285,803 bp, 3289 and 3323 CDS, 48 and 49 tRNA genes, the same number of rRNA (3) genes, 583 and 586 hypothetical proteins, 2697 and 2726 functional proteins for *B. abortus* and *B. melitensis* respectively. *B. abortus* strains exhibit a similar number of candidate genes, while *B. melitensis* strains showed some differences, especially in the SRR19520422 Faiyum strain. Also, *B. melitensis* clarified differences in antimicrobial resistance genes (KatG, FabL, MtrA, MtrB, OxyR, and VanO-type) in SRR19520319 Faiyum and (Erm C and Tet K) in SRR19520422 Faiyum strain. Additionally, the whole genome phylogeny analysis proved that all *B. abortus* strains were related to vaccinated animals and all *B. melitensis* strains of Menoufia clustered together and closely related to Gharbia, Dameitta, and Kafr Elshiek. The Bowtie2 tool identified 338 (eight *B. abortus*) and 4271 (eighteen *B. melitensis*) single nucleotide polymorphisms (SNPs) along the genomes. These variants had been annotated according to type and impact. Moreover, thirty candidate genes were predicted and submitted at GenBank (24 in *B. abortus*) and (6 in *B. melitensis*). This study contributes significant insights into genetic variation, virulence factors, and vaccine-related associations of *Brucella* pathogens, enhancing our knowledge of brucellosis epidemiology and evolution in Egypt.

**Supplementary Information:**

The online version contains supplementary material available at 10.1007/s00239-024-10173-0.

## Introduction

*Brucella* is a stealth pathogen that evades immune response and causes brucellosis which is regarded as a zoonotic infectious disease all over the world (Corbel 1997). The *Brucella* genus now includes twelve species that infect various domestic and wild-life animal species (Dawood et al. 2023). Six *Brucella* species have been identified as correlating to their pathogenicity and natural hosts. They are *Brucella abortus* (cattle), *Brucella melitensis* (sheep and goats), *Brucella canis* (dogs), *Brucella suis* (pigs), and *Brucella neotomae* (desert wood rat), *Brucella ovis* (rams) (Whatmore 2009; Blasco and Molina-Flores 2011). In Egypt, *B. melitensis* biovar 3 is the most common cause of animal and human infection, subsequent to *B. abortus* biovar 1 (Hamdy and Zaki 2018). Regardless of the implantation brucellosis control strategy in 1981, it remains widespread in Egypt (Hegazy et al. 2021).

*Brucella’s* genome is circular and very stable, and its size is 3.3 Mb (approximately 2.1 Mb on chromosome I and 1.2 Mb on chromosome II) (Michaux et al. 1993). There are striking similarities between *B. abortus* and *B. melitensis* corresponding to the homology degree found in DNA-DNA hybridization studies (Wang et al. 2012). Whole-genome sequencing (WGS) is regarded as the perfect technology for studying genomic variants of organisms in depth; it has become simple, cost-effective, and widely available (Tan et al. 2015).

Bioinformatics has significant roles not only in dealing with massive amounts of data, but also in the prediction, analysis, or interpretation of clinical and preclinical results, drug discovery, evaluation, and development (Khan et al. 2022). The comprehensive genome analysis provides detailed insights into genome quality evaluation, a phylogenetic tree, subsystem overview, antimicrobial resistance (AMR) genes, and candidate genes (Wang et al. 2020). Hence, in this study, we carried out detailed genetic characterization and comparative genomics analysis of some available Egyptian strains.

## Materials & Methods

### Retrieve Data

The sequences of *B. abortus* and* B. melitensis* strains in Egypt (from 2007 to 2019) were obtained from the National Center for Biotechnology Information (NCBI) repository and downloaded from European Nucleotide Archive (ENA) in fastq format (Tables S1 and S2). Most of the genomic analysis tools used in this study were done at the Bacterial and Viral Bioinformatics Resource Center (BV-BRC) server. Therefore, comparative genomics of available Egyptian strains (*B. abortus* *n* = 8 and *B. melitensis* *n* = 18) have been used in the current research to determine their closeness with vaccine strains (RB51 and Rev.1) for *B. abortus* and *B. melitensis* respectively.

### Assembly and Annotation of Genomes

The processed fastq files are applied to the PATRIC assembly service which has seven different strategies. A Unicycler (V 0.4.8) pipeline used for the genome assembly of bacterial and archaeal genomes and the “AUTO” selection was applied to assemble the paired read to short read contigs. The unicycler employs the SPAdes de novo assembler to construct an initial assembly graph and subsequently refines by incorporating information from both short and long reads to generate high-quality, circular sequences and resolves complex repeat structures (Wick et al. 2017). A bandage program (V 0.8.1) was employed for visualization of these de novo assembly contigs. It primarily helps to identify errors and structure rearrangements in next-generation sequencing (NGS) data (Wick et al. 2015). Pilon (V 1.23) was used for correcting assembly errors for short reads to enhances the quality of draft genome assemblies producing more contiguous and accurate genome assemblies with better gene identification with remarkable accuracy in the identification of both small and large sequence variants (Walker et al. 2014). Additionally, TrimGalore was used for trimming the reads. It enables the automation of adapter trimming, and the removal of low-quality reads to enhance the quality of sequencing data. Also, SAMtools with default parameters were employed for reading mapping (Li et al. 2009). Finally, QUAST Version 5.0.2 is used for the automated and comprehensive analysis of genomics sequence assemblies to check the quality and completeness of the assembly output (Gurevich et al. 2013). Genome annotation refers to the process of locating functional components along the sequence of a genome. To annotate genomic characteristics in *Brucella* strains, rapid annotation using a subsystems technology tool kit (RASTtk) in BV-BRC was utilized (Aziz et al. 2008).

### Comprehensive Genome Analysis

Comprehensive Genome Analysis was applied using the meta-service on the BV-BRC server that acquires raw data or the single or paired read to calculate genome assembly, annotation, quality control, AMR, and candidate genes that are specific for important functions (Davis et al. 2019).

### Alignment Analysis

Progressive Mauve application was used to perform Multiple Sequence Alignment (MSA). Using MAUVE, the visually observable locally collinear blocks (LCBs), which are identified by specific color codes, were used to locate the genomic areas that were shared by all the matched sequences. LCBs indicate the homologous sequence found in two or more genomes without rearrangement (Darling et al. 2010). *B. abortus *RB51-AHVLA (Taxonomy ID: 1198700) and *B. melitensis* strain Rev.1 (Genome ID: 29459.409) were used as reference genomes from the PATRIC database. Moreover, at the level of Menoufia Governorate, *Brucella* from different centers was aligned with reference vaccine genome Rev.1.

### Phylogenetic Analysis

Performing phylogenetic relationships in bacterial populations is critical to understanding the molecular evolutionary chronicle (O’Callaghan and Whatmore 2011). We used a BV-BRC phylogenetic tree called “Codon Trees” to know the evolutionary relationship between our strains and other outgroup strains to give an accurate view. It makes use of the predefined PATRIC protein global families (PGFams) (Davis et al. 2016), choosing 10–1000 single-copy families from among members of a genomic group. Alignments are produced for protein sequences of every family using Muscle (Edgar 2004), and for their corresponding nucleotide sequences, BioPython’s codon-align function was employed (Cock et al. 2009). To create confidence values, 100 rapid bootstrap rounds are executed within RaxML (Stamatakis et al. 2008).

### Comparative Genomic Analysis

The comparative genome analysis was executed at the BV-BRC server. It combines two PATRIC tools (Protein Family Sorter and Comparative Pathways Viewer) as well as subsystems (Overbeek 2005; Overbeek et al. 2014). All three systems use two protein families: the local families (PLFams) for intra-genus comparisons and the global families (PGFams) for cross-genus comparisons (Davis et al. 2016). The PATRIC Protein Family Sorter tool is utilized to investigate the distribution patterns of protein families with different options: (1) present in all families to find the core genome, (2) absence from all families to examine the accessory genome and 3) mixed/either option which is the default is to determine the pangenome.

### Variation Analysis

The variation analysis tool on the BV-BRC server finds the alterations from the reference (*B. abortus* RB51-AHVLA (Taxonomy ID: 1,198,700) and *B. melitensis* strain Rev.1 (Genome ID: 29,459.409)) and identifies single nucleotide variants (SNVs) and short insertions/deletions (indels) from aligned NGS data. Numerous tools have been developed such as BWA-mem and BWA-mem-strict (Li 2013), Bowtie2 (Langmead and Salzberg 2012), and LAST (Frith et al. 2010). FreeBayes (Garrison and Marth 2012) and SAMtools (Li et al. 2009) are the two SNP callers. Nevertheless, even minor variations could result in a significant number of variant calls across the entire genome. Therefore, it is crucial to select a reliable variant caller for both SNVs and indels and further evaluate and refine it to achieve maximum efficiency when analyzing the data. The analysis was done using the four different aligners.

### Gene Prediction

The assembled contigs files generated from the previous steps from the BV-BRC website were subjected to various gene prediction servers: GeneMark.hmm prokaryotic (version 3.25), GeneMarkS-2 (Lomsadze et al. 2018), and EasyGene 1.2 Gene finding in prokaryotes (Larsen and Krogh 2003). To find the most concealed state sequence, the GeneMark.hmm server utilizes a hidden Markov model (HMM) architecture and the Viterbi algorithm, while GeneMarkS-2 is an ab initio technique that finds species-specific (native) genes using a model generated from self-training, as well as heuristic models meant to identify horizontal gene transfers. To evaluate GeneMarkS-2’s precision, the identified genes were confirmed through Clusters of Orthologous Groups (COG) proteomics experiments, annotation and N-terminal protein sequencing. EasyGene is also based on an algorithmic estimation of an HMM for an entirely new genome. After identifying the putative genes, they are evaluated using the HMM score and according to their scores and the length of the open reading frame (ORF), the statistical significance of the genes is calculated.

### Molecular Evolutionary, Phylogenetic Analysis, and Conserved Regions of Predicted Genes

The 30 predicted genes underwent BLASTN and multiple sequence alignments from various genotypes via ClustalW multiple alignments impeded in BIOEDIT 7.2 software. The conserved regions refer to parts of DNA or protein sequences that have been preserved during evolution and contain functional elements such as protein-coding, non-coding regulatory domains, or structural motifs. In DNA sequences, the terms “similarity” and “identity” are used interchangeably to describe the degree of resemblance between two sequences, with identity specifically indicating that the two sequences are identical in that region. High sequence identity between regions of DNA typically indicates conservation, as these regions have been preserved across multiple organisms and may have important functional implications. These regions were identified by comparing sequences from multiple organisms or individuals. The “find conserved regions” option in the BIOEDIT 7.2 software with the default parameters of minimum length = 15 and max average entropy = 0.2. was applied to detect the conserved regions in the thirty predicted genes. These parameters ensure that identified regions meet a certain level of similarity and length to be considered conserved (Zhang et al. 2000).

After that, MEGA11 software and the ClustalW tool that impeded in it were employed for the phylogenetic analysis of the thirty predicted genes (Tamura et al. 2021). The multiple sequence alignment was performed using MAFFT server which offers various multiple alignment strategies. They are classified into three types, (a) the progressive method, (b) the iterative refinement method with the WSP score, and (c) the iterative refinement method using both the WSP and consistency scores. In order to obtain more accurate alignments, three new options, L-INS-i, G-INS-i, and E-INS-i, were used to align sequences globally using the Needleman-Wunsch algorithm. The five different constructed phylogenetic tree methods in MEGA11 software were used. Not only computing pairwise distances and overall mean distances but also different models were assessed by MEGA11 software. The best model selection “find best DNA/protein models” option was applied using MEGA to justify the choice of model for phylogenetics. Then the phylogenetic analysis was conducted on nucleotide substitution type and choice of the best model General Time Reversible Model (GTR). The GTR model is designed for inferring evolutionary relationships from DNA sequence data by accounting for different rates of nucleotide substitutions and the reversibility of these processes over time, making it an accurate tool for phylogenetic analysis. The initial tree(s) for the heuristic search were conducted automatically by applying Neighbor-Join and BioNJ algorithms to a matrix of pairwise distances estimated using bootstrap methods, and the GTR model and then selecting the topology with superior log likelihood value. Phylogenetic trees use bootstrap values to measure the confidence level of sequence-based phylogeny on a scale of 1 to 100.

## Results

### Genome Assembly and Annotation

A comprehensive genome analysis of two *Brucella (B.)* species in Egypt was reported. The genome assembly of the eight *B. abortus* strains showed almost similar characteristics; contigs average were (30–35), genome length of 3,250,377 bp, and G + C content of 57.26%. The N50 length is 246,321 bp, which is recognized as the shortest sequence length at 50% of the genome. The L50 count was 6, which is known as the minimum number of contigs whose length summation yields N50 (Table 1). The *B. melitensis* strains were estimated with different numbers of contigs with an average of 26 in most of the strains (Dakahilia, Aswan, Ismailia, Beheira, and Beni-suef) and high numbers in (Asyut 40, Giza 42, and Beheira 58), an average of estimated genome length was 3,285,803 bp and G + C content of 57.25%. Also, the N50 length was 260,369 bp, and also the L50 was 4 (Table 2).

**Table 1:** Genome assembly & annotation of *B. abortus* using BV-BRC server

	Genome assembly					Genome features			Protein features						
Strains	Contigs	Contig L50	Contig N50	GC Content	Genome Length (bp)	CDS	tRNA	rRNA	Hypothetical proteins	Functional Proteins	Proteins have an EC number	Proteins have GO	Proteins with Pathway	PLfam	PGfam
SRR12368029 Giza	27	5	308,645	57.25	3,255,378	3286	49	3	585	2701	938	804	720	3,197	3224
SRR12368032 Qalyubia	31	6	207,875	57.26	3,253,665	3293	49	3	594	2699	938	804	720	3,194	3223
SRR12368034 Menoufia	35	5	259,517	57.26	3,249,471	3276	48	3	579	2697	939	805	721	3,183	3211
SRR12368035 Dakhilia	30	5	250,166	57.25	3,256,265	3289	49	3	584	2705	939	805	721	3,192	3223
SRR12368050 Beheira	27	5	308,645	57.25	3,254,910	3282	47	3	583	2699	938	804	720	3,187	3215
SRR12368036 Beheira	43	7	177,813	57.28	3,247,651	3283	48	3	589	2694	938	804	720	3,185	3212
SRR12368049 Beheira	36	6	207,986	57.26	3,249,076	3281	48	3	583	2698	940	806	722	3,185	3214
SRR12368047 Asyut	30	6	249,923	57.26	3,250,377	3289	48	3	592	2693	939	805	721	3,190	3220

**Table 2:** Genome assembly & annotation of *B. melitensis* using BV-BRC server

	GenomeAssembly					GenomeFeatures			ProteinFeatures						
Strains	Contigs	Contig L50	Contig N50	GC Content	Genome Length (bp)	CDS	tRNA	rRNA	Hypothetical proteins	Functional Proteins	Proteins have an EC number	Proteins have GO	Proteins with Pathway	PLfam	PGfam
SRR19520420 Dameitta	25	3	530,063	57.25	3,285,821	3312	49	3	585	2727	948	813	730	3213	3239
SRR19520320 Sharqia	25	4	249,819	57.24	3,285,196	3316	49	3	589	2727	949	814	731	3206	3232
SRR19520411 Menoufia	26	4	249,860	57.25	3,285,803	3323	49	3	597	2726	949	814	731	3213	3239
SRR19520326 Matruh	29	4	249,875	57.25	3,285,027	3310	49	3	584	2726	949	814	730	3205	3231
SRR12368031 Qalyubia	34	5	207,793	57.25	3,281,132	3318	48	3	596	2722	951	816	731	3210	3236
SRR19520382 Kafr_Elshiek	25	4	249,814	57.25	3,285,789	3308	49	3	586	2722	949	814	730	3207	3233
SRR19520361 Gharbia	26	4	251,059	57.25	3,285,969	3317	49	3	589	2728	949	813	731	3210	3236
SRR19520319 Faiyum	29	3	249,145	57.25	3,313,423	3347	49	3	589	2728	950	815	731	3220	3239
SRR19520422 Faiyum	29	4	249,809	56.89	3,328,336	3380	49	3	641	2739	950	815	731	3226	3288
SRR19520421 Dakahlia	26	4	249,860	57.25	3,285,419	3311	49	3	583	2728	949	814	730	3208	3234
SRR12368033 Menoufia	34	6	207,715	57.26	3,291,668	3320	49	3	591	2735	949	814	731	3219	3254
SRR12368040 Giza	42	8	141,823	57.24	3,283,305	3311	49	3	589	2731	952	817	732	3219	3244
SRR12368037 Ismailia	29	5	246,975	57.25	3,284,605	3311	49	3	586	2725	950	815	732	3207	3233
SRR12368024 Beheira	28	5	247,341	57.25	3,285,197	3304	49	3	579	2725	948	813	730	3203	3230
SRR12368042 Beni_Suef	28	5	247,280	57.25	3,285,151	3312	49	3	586	2726	949	814	731	3212	3237
SRR19520334 Aswan	26	4	249,880	57.24	3,285,43	3307	49	3	583	2724	948	813	730	3206	3231
SRR12368048 Asyut	40	6	186,636	57.25	3,279,118	3318	48	3	593	2725	953	817	732	3211	3238
SRR12368025 Beheira	58	10	129,247	57.29	3,273,712	3310	48	3	586	2724	952	817	732	3207	3232

**Fig. 1:**
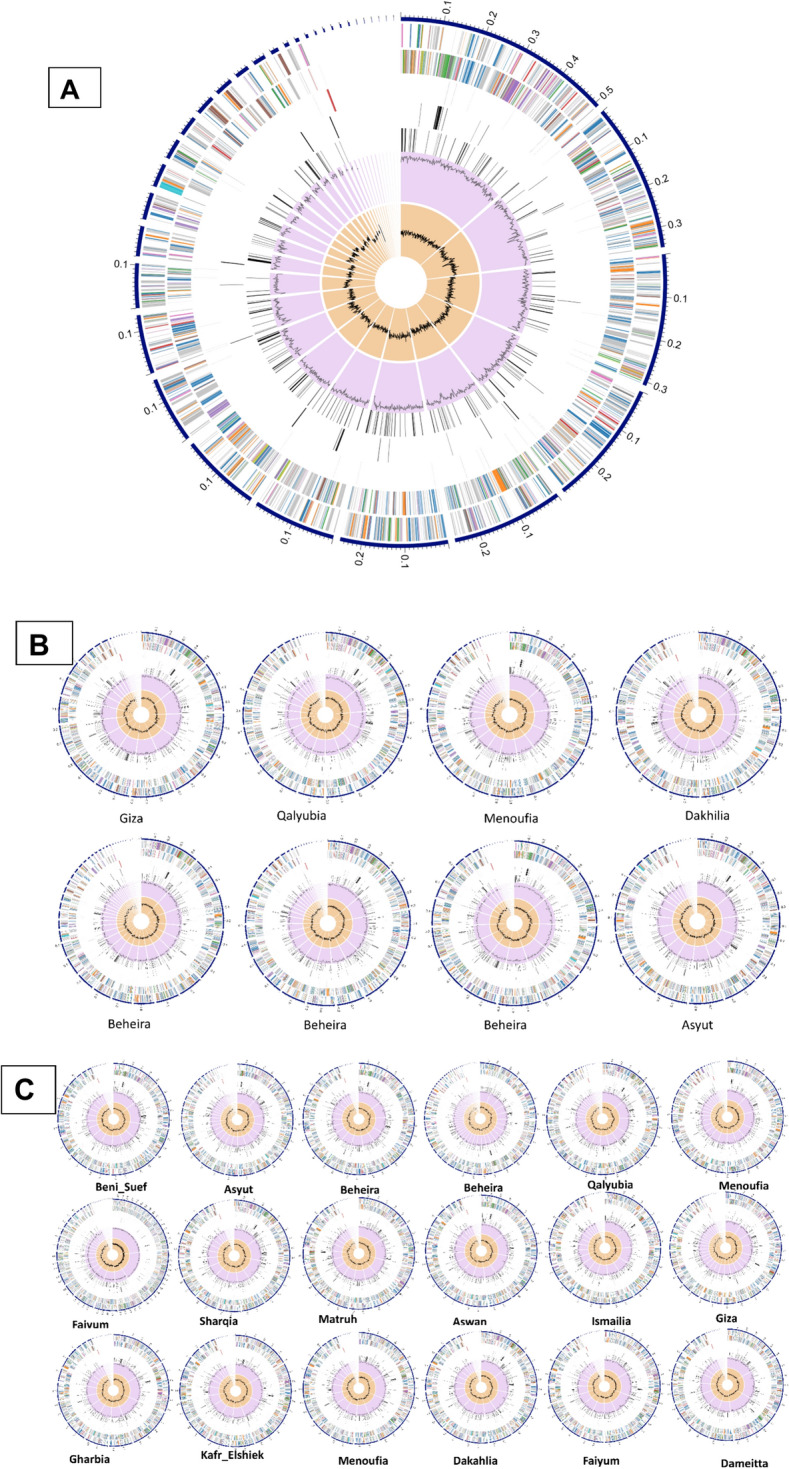
The circular genome of *Brucella* (**A**) is illustrated, along with the circular genomes of eight *B. abortus* (**B**) and eighteen *B. melitensis* (**C**). The figure represents various genomic annotations, arranged from outside to inside, such as contigs, CDS on the forward and reverse strands, tRNA, CDS of AMR genes, CDS of virulence factor, GC content, and GC skew. The colors of CDS on the two strands reflect the subsystem to which these genes belong (see Subsystems below)

The genome annotation of *Brucella* species had an average of 3289 and 3323 coding DNA sequence (CDS), 48 and 49 transfer RNA genes (tRNA), 3 ribosomal RNA genes (rRNA), 583, 586 hypothetical proteins, and 2697, 2726 functional proteins in *B. abortus and B. melitensis* respectively (Fig. 1). These functional proteins estimated an average of 939 proteins have Enzyme Commission (EC) numbers, 805, 814 have Gene Ontology (GO), and 721, 731 proteins were linked to KEGG pathways in *B. abortus and B. melitensis* respectively. PATRIC annotation possessed 3190 and 3213 PLFams and 3220, and 3239 PGFams proteins for *B. abortus* and *melitensis* respectively (Tables 1 and 2).

### Candidate Genes and Antimicrobial Resistance Genes

Numerous annotated genes in *B. abortus* exhibit similarities with well-known genes associated with antibiotic resistance, transporters, drug targets as well as virulence factors, as indicated in (Table 3), while the AMR patterns in (Table 4) were consistent across all eight strains.

**Table 3:** Candidate genes of eight *B. abortus* strains

	Source	Genes
Antibiotic resistance	CARD	2
	PATRIC	33
Transporter	TCDB	23
Drug target	DrugTarget	4
	TTD	1
Virulence factor	PATRIC_VF	1
	VFDB	44
	Victors	225

**Table 4:** Antimicrobial resistance genes of the two *Brucella* species strains

Species	Genes	AMR mechanism
*B. abortus*	FosX	Antibiotic inactivation enzyme
	Alr, Ddl, S10p, S12p, EF-G, inhA, EF-Tu, Iso-tRNA, folA, Dfr, kasA, folP, rho, MurA, rpoB, rpoC, fabI, gyrA, and gyrB	Antibiotic candidate in susceptible species
	TriABC-OpmH, MacA, MacB	Conferred antibiotic resistance via the efflux pump
	gidB	Absence of the gene causes resistance
	PgsA, GdpD	Antibiotic resistance is gained via protein-altering cell wall charge
	OxyR	Regulator of antibiotic resistance gene expression
*B. melitensis*	**KatG only in SRR19520319Faiyum**	**Enzyme for antibiotic activation**
	FosX	Enzyme for antibiotic inactivation
	Alr, Ddl, S10p, S12p, EF-G, inhA, EF-Tu, Iso-tRNA, folA, Dfr, kasA, folP, rho, MurA, rpoB, rpoC, fabI, gyrA, and gyrB	Antibiotic candidate in susceptible species
	**Erm(C) only in SRR19520422Faiyum**	**Antibiotic target modifying enzyme**
	**FabL-like only in SRR19520319Faiyum**	**Antibiotic target replacement protein**
	TriABC-OpmH, MacA, MacB, **Tet(K) only in SRR19520422 Faiyum**	**Conferred antibiotic resistance via the efflux pump**
	gidB	Absence of the gene causes resistance
	PgsA, GdpD,	Antibiotic resistance is gained via protein-altering cell wall charge
	OxyR **OxyR, MtrA, MtrB, VanO-type only in SRR19520319 Faiyum**	**regulator of antibiotic resistance gene expression**

The bold is significant to the Genes and the AMR mechanism that are different from the other strains of *B. melitensis*

While the 18 *B. melitensis* strains showed some differences, especially in the SRR19520422 Faiyum strain in candidate genes (Table 5). Otherwise, SRR19520319 Faiyum and SRR19520422 Faiyum strains revealed differences in antimicrobial resistance genes comprising antibiotic activation enzyme (KatG), antibiotic target replacement protein FabL-like and regulator of antibiotic resistance gene expression (MtrA, MtrB, OxyR, VanO-type) for SRR19520319 Faiyum strain, and altering antibiotic target enzyme (Erm C) and an efflux pump that confers antibiotic resistance (Tet K) for SRR19520422 Faiyum strain (Table 4).

**Table 5:** Candidate genes of eighteen *B. melitensis* strains

Strain	Antibiotic Resistance			Transporter	Drug Target		Virulence Factor		
	CARD	TCDB	NDARO	TCDB	DrugBank	TTD	PATRIC_VF	VFDB	Victors
SRR19520420 Dameitta	2	33	–	25	4	1	1	44	223
SRR19520320 Sharqia	2	33	–	25	4	1	1	44	223
SRR19520411 Menoufia	2	33	–	25	4	1	1	44	223
SRR19520326 Matruh	2	33	–	25	4	1	1	44	224
SRR12368031 Qalyubia	2	33	–	25	4	1	1	44	223
SRR19520382 KafrElshiek	2	33	–	25	4	1	1	44	223
SRR19520361 Gharbia	2	33	–	25	4	1	1	44	223
SRR19520319 Faiyum	2	33	–	25	6	1	1	44	223
**SRR19520422 Faiyum**	**6**	**36**	**4**	**26**	**6**	**1**	1	44	223
SRR19520421 Dakahlia	2	33	-	25	4	1	1	44	223
SRR12368033 Menoufia	2	33	-	25	4	1	1	44	223
SRR12368040 Giza	2	33	-	25	4	1	1	44	223
SRR12368037 Ismailia	2	33	-	25	5	1	1	44	223
SRR12368024 Beheira	2	33	-	25	4	1	1	44	223
SRR12368042 BeniSuef	2	33	-	25	4	1	1	44	223
SRR19520334 Aswan	2	33	-	25	4	1	1	44	223

The bold is significant to the different number of candidate genes of SRR19520422 Faiyum from the other *B. melitensis*

From the above results, the comprehensive genome analysis of the two species of *Brucella* and between each species and its strain provide striking resemblance in the genome and protein feature except slightly differences which proven the stability of *Brucella* species genome. However, the AMR genes profile displayed some novel genes that were not identified or with limited known to *Brucella* which may acquire from other species microorganisms.

### Subsystems in *B. abortus *and *B. melitensis*

**Fig. 2:**
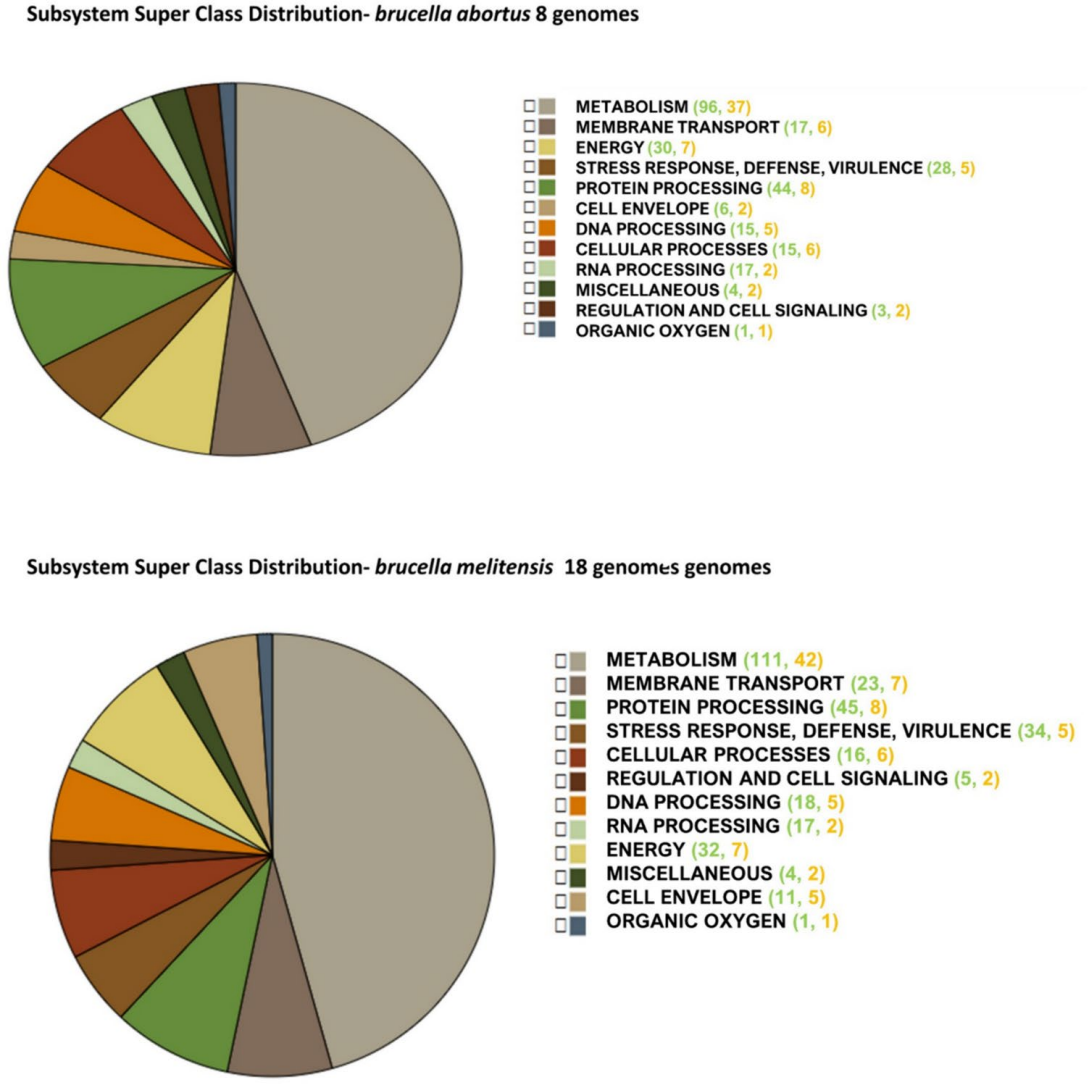
Distribution of subsystem category for *B. abortus* and *B. melitensis* strains which are organized via this pattern Subsystem Counts (Subsystems, Genes)

A subsystem is a collection of proteins that work together to carry out a certain biological process or structural complex. The pie chart summarizes the features of each subsystem and their coverage. A total of 83, 92 genes have been allocated to several subsystems, with the metabolism of amino acids receiving the most (37, 42). It was organized according to this pattern of Subsystem Counts (Subsystems, Genes), which means that in the case of *B. abortus* metabolism, it is a specific biological process with 96 subsystems controlled by 37 genes (Fig. 2).

### Comparative Genome Analysis

#### Genome Alignment

**Fig. 3:**
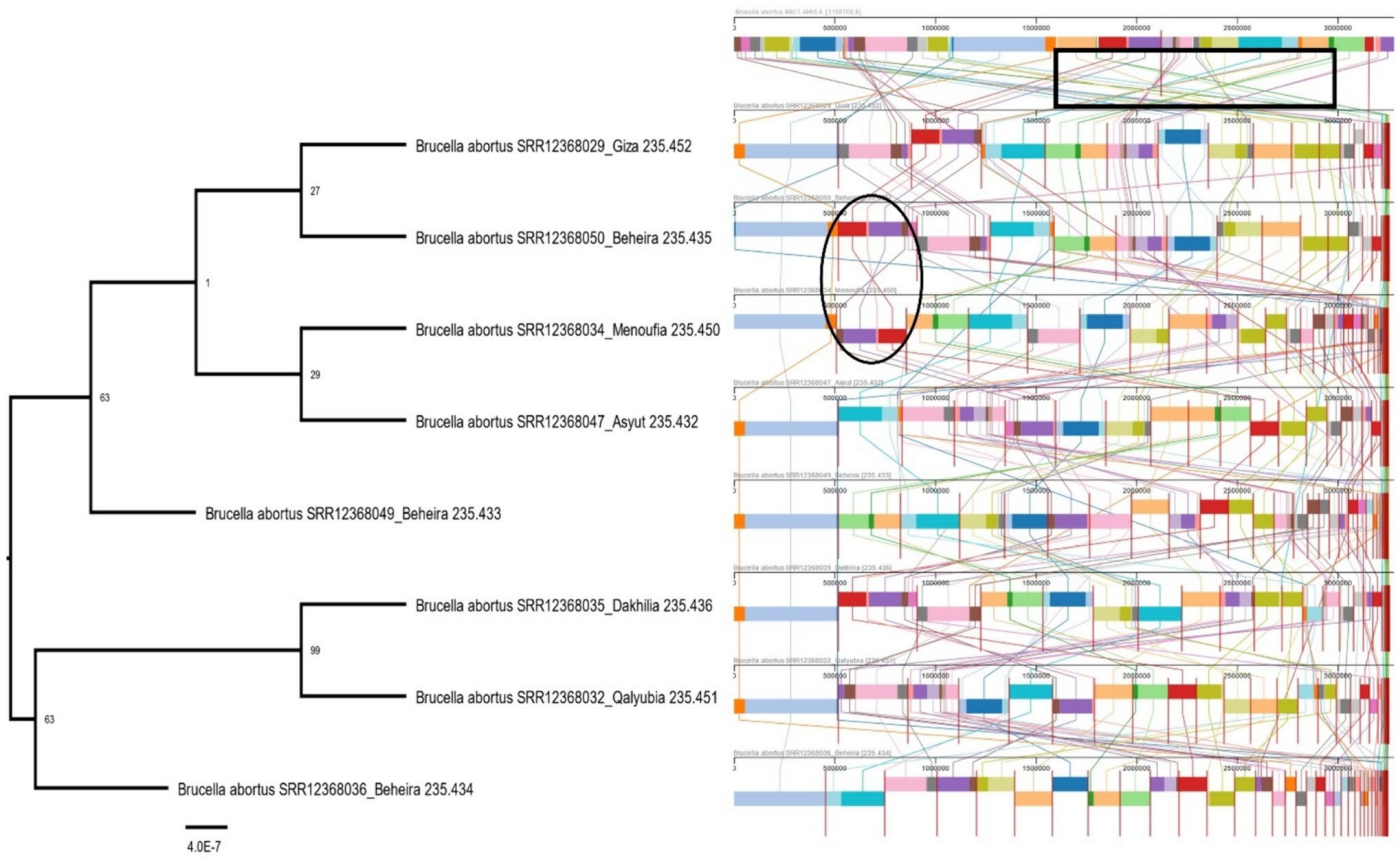
The multiple sequence alignment of *B. abortus* is illustrated. The black circle represents the inversion region between two genomes of *B. abortus*, while colored lines between genomes in black boxes represent rearrangements of genomic sequences. The whole genome phylogeny (eight genomes) of *B. abortus* which was built on the protein and gene sequences for those 1000 genes performed at the PATRIC server confirmed the relatedness of *B. abortus* strains. The length of a branch is relative to the total number of changes at each site

**Fig. 4:**
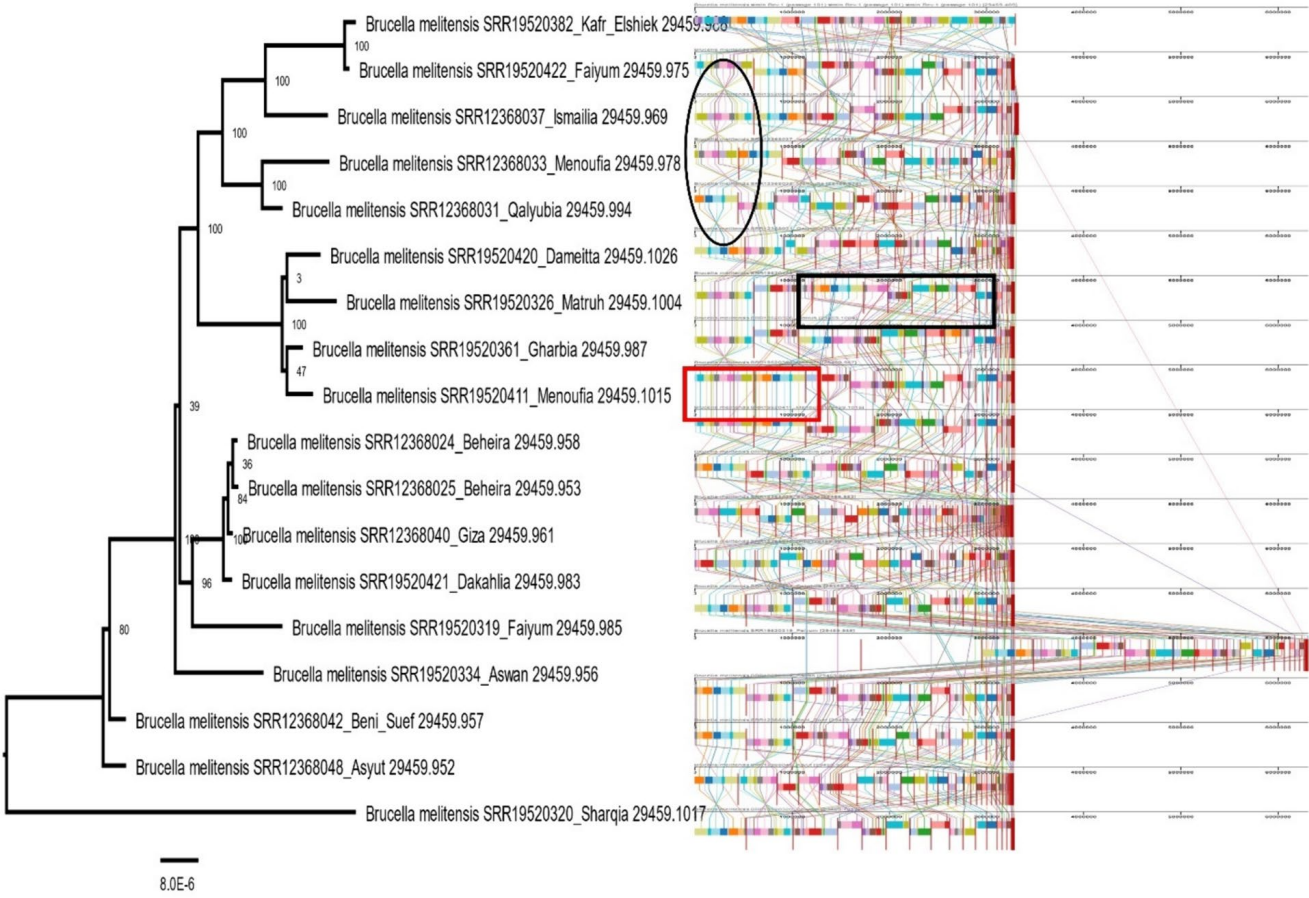
The multiple sequence alignment of *B. melitensis* alignment is displayed. The black circles represent the inversion regions between the some of genomes of *B. melitensis*. The red box represented the same arrangement of the genomic sequences, while colored lines between genomes in the black box represented rearrangements of the genomic sequences. The phylogenetic tree for 18 whole genomes of *B. melitensis* which was built on the protein and gene sequences for those 1000 genes performed at PATRIC server confirmed the relatedness of *B. melitensis* strains. The length of a branch is relative to the total number of changes at each site (Color figure online)

**Fig. 5:**
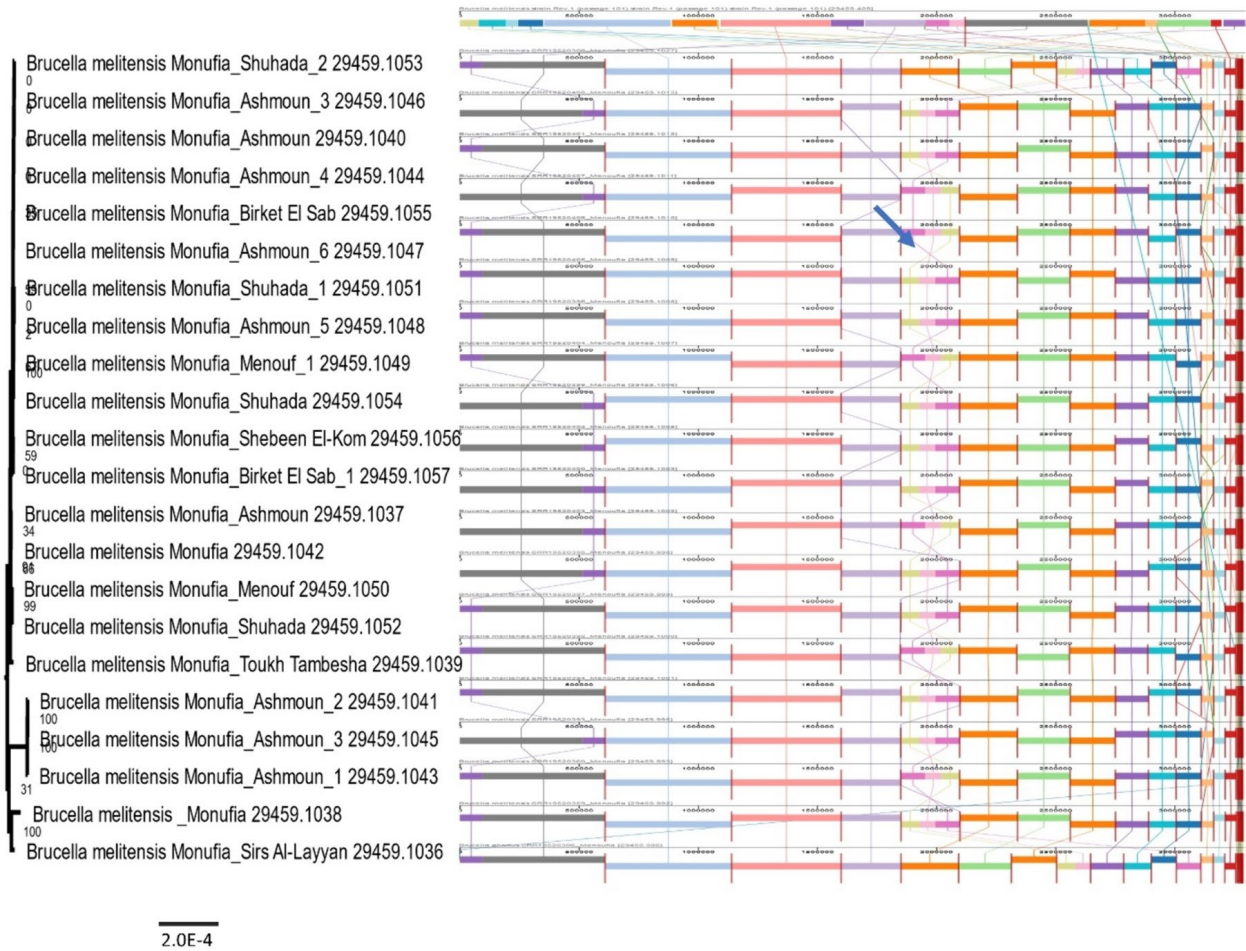
The multiple sequence alignment of *Brucella* species is illustrated at Menoufia governorate centers. There is no rearrangement and inversion between the different *B. melitensis* species except for some slight inversions at the regions pointed by the arrow. The figure showed the conservation regions in different *B. melitensis* species at Menoufia centers. The phylogenetic tree for 22 whole genomes of Menoufia governorate from different centers which was built on the protein and gene sequences for those 1000 genes performed at the PATRIC server confirmed the close relatedness of *B. melitensis* strains. The length of a branch is relative to the total number of changes at each site

Multiple sequence alignments of eight and eighteen genomes of* B. abortus* and* B. melitensis* with their reference vaccine genomes (RB51 and Rev.1) respectively were illustrated in (Figs. 3 and 4) using the progressive Mauve alignment program. Each genome is put out horizontally on a black horizontal center line that contains its name. Colored blocks represented homologous segments, while colored lines between genomes denote rearrangements or inversions (s-shape). Blocks positioned above the center line signify alignment in the forward direction of the initial genomic sequence, while those below indicate alignment in the reverse complement (inverse) direction. Moreover, at the level of Menoufia Governorate, *Brucella* strains from different centers are aligned with the reference vaccine genome Rev.1 illustrated in (Fig. 5).

#### Phylogenetic Analysis

**Fig. 6:**
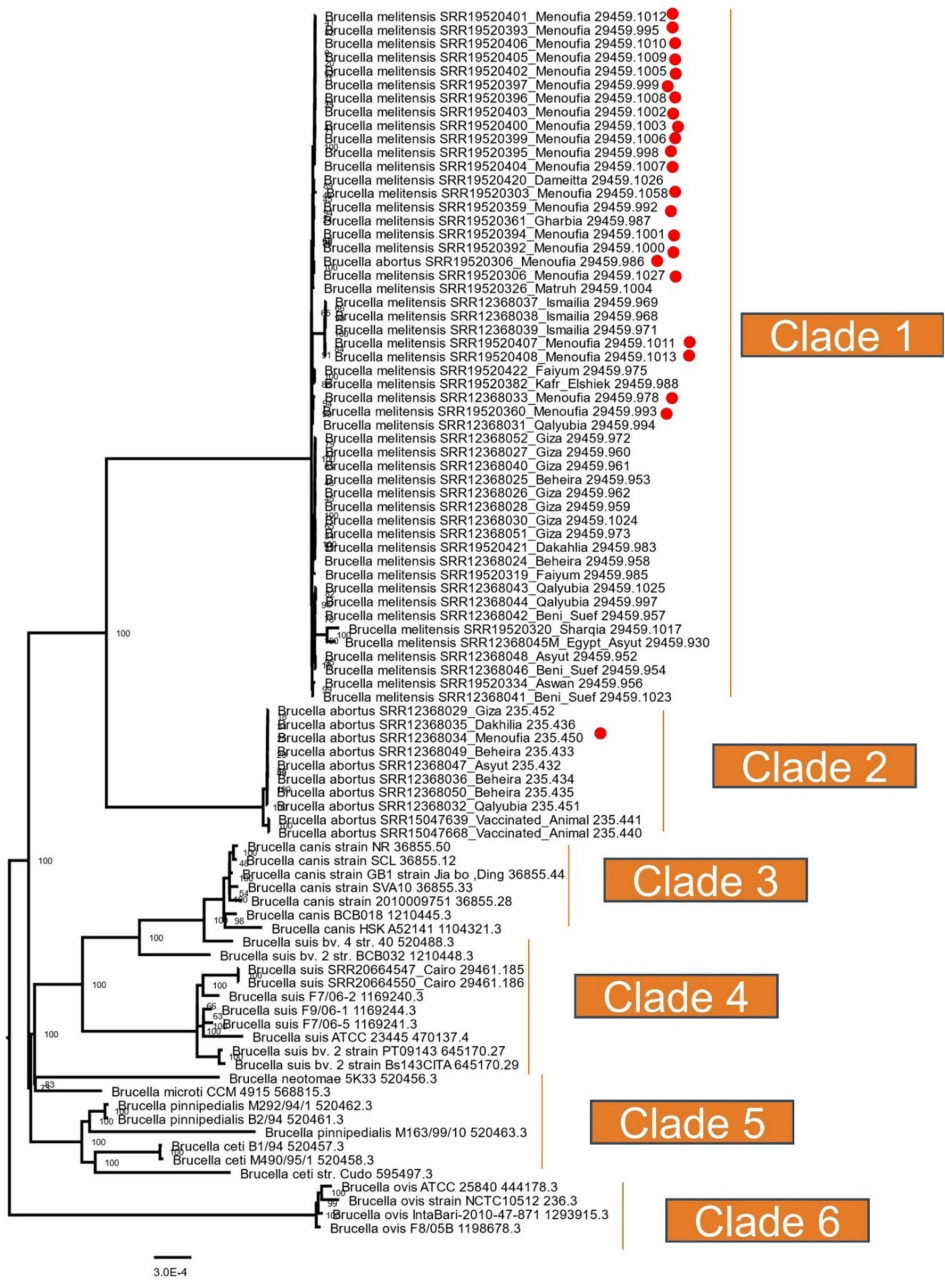
The whole genome phylogeny (90 genomes) involving Egyptian strains (*n* = 26) with other available Egyptian and global strains is shown. The tree was built on the protein and gene sequences for those 1000 genes performed at the PATRIC server. Branch support values are obtained using 100 bootstrap and the length of a branch is relative to the total number of changes at each site

We constructed a whole genome phylogeny (90 genomes) involving Egyptian strains (*n* = 26) along with other available Egyptian and global strains. The tree was built on the protein and gene sequences for those 1000 genes. The whole genome phylogeny is clustered into six clades. The phylogeny indicated that all Egyptian *B. abortus* and *B. melitensis* strains are closely distance from each other. All *B. melitensis* strains of Menoufia clustered together and closely related to Gharbia, Dameitta, and Kafr Elshiek. On the opposite hand, *B. melitensis* stains of Giza were closely related to Beheira and Dakahlia. Also, *B. abortus* strains were related to vaccinated animals. *B. Suis* strains of Cairo were strictly related to *B. Suis* F7/06–2 from Germany. When compared to other *B. abortus* and *B. melitensis* strains, *B. canis*, *B. ovis*, *B. Ceti*, *B. suis*, *B. neotomae*, *B. microti*, and *B. pinnipedialis* strains were used as outgroups (Fig. 6).

#### Core and Accessory Gene Compositions

Pangenome refers to the totality of shared and distinct genetic material present in a particular species that combines the genetic information of all the genomes sampled. It helps to better understand the comprehension composition and evolution of gene content within a population. The core genome is the identification of protein families that are either conserved across all the genomes, and that are conserved only in specific subsets of selected genomes are the accessory proteome.

In the case of *B. abortus* (8 genomes) and *B. melitensis* (18 genomes), the pan-genome analysis revealed 2677 and 2650 core genes and zero accessory genes of 2742 and 3911 gene pan-genome respectively. It is indicated that the genomes of these two *Brucella* species are highly similar, and they share most of their genetic material. The absence of accessory genes in the pan-genome analysis indicates that there is little genetic diversity among the sampled strains of these two species as shown in Fig. 6.

#### Single Nucleotide Polymorphism (SNPs)

Using the Bowtie2 tool identified 338 SNPs in all eight *B. abortus* genomes against RB51-AHVLA vaccine strain (reference genome) and from these SNPs 198 were commonly identified in all read libraries that we removed. The variants were annotated according to their possible effect and location on coding sequences (CDSs) via SnpEff (Cingolani et al. 2012). Variants type of classification exhibited 95 insertion variants, 28 nonsynonymous variants, 58 synonym variants, and 6 deletion variants. There were 26 frameshifts predicted, with 11 in-frame insertions 1 disrupted in-frame deletion, 37 intergenic regions, and 28 missense variants. Also, variants impact counted 53 modifiers, 40 moderate, 9 low, and 14 high.

In the case of *B. melitensis*, 4271 SNPs were identified in all eighteen genomes against the REV1 vaccine strain, and from these SNPs 2725 were commonly shared in all read libraries that we ignored. Also, variants type of classification revealed 394 insertion variants, 1831 nonsynonymous variants, 2229 synonym variants, and 200 deletion variants. Moreover, there were 230 frameshifts predicted, with 50 in-frame insertions, 20 in-frame deletions, 10 disrupted in-frame insertions, 5 disrupted in-frame deletions, and 773 intergenic regions, 1780 missense variants, 19 stop lost, 31 stop gained, 3 stop retained variants, 8 conservatives in-frame insertion, 8 conservatives in-frame deletion and 15 stop lost & splice region variants. In addition, variants impact counted 778 modifiers, 1850 moderate, 1126 low, and 228 high.

### Conserved Regions, Molecular Evolutionary and Phylogenetic Analysis of Predicted Genes

#### Conserved Regions

The gene’s predicted servers (GeneMark.hmm prokaryotic (version 3.25), GeneMarkS-2, and EasyGene 1.2 Gene finding in prokaryote) were used to predict *B. abortus* (SRR12368034) and *B. melitensis* (SRR12368033). They revealed 1937 predicted genes for *B. abortus* and 1448 for *B. melitensis*. Hence, we submitted the most similar query genes to database sequence (24) for *B. abortus* and (6) for *B. melitensis* and accession numbers were obtained for the submitted genes. Using BIOEDIT for MSA, each gene has different numbers of conserved regions, segment length, and average entropy (Hx) (Tables 6 and 7).

**Table 6:** The predicted genes with accession numbers and conserved regions of submitted genes of *B. abortus* using BIOEDIT

Species	Isolate	Gene	Length (bp)	Accession Numbers	Number of conserved regions	Average Entropy (Hx)
*B. abortus*	AE-1	ssb	507	LC742715	6	0.0000
	AE-1	Tuf-2	1176	LC743623	1	0.0018
	AE-1	pth	753	LC743839	2	0.0000
	AE-1	yibK	1869	LC743840	26	0.0000
	AE-1	OMP31b	786	LC743841	7	0.0000
	AE-1	BCSP31	1059	LC743842	12	0.0000
	AE-1	VirB3	351	LC743474	4	0.0000
	AE-1	VirB1	717	LC743475	8	0.0000
	AE-1	VirB8	720	LC743476	11	0.0000
	AE-1	VirB11	1086	LC743477	15	0.0000
	AE-1	VirB4	2496	LC743478	30	0.0000
	AE-1	VirB6	1044	LC743479	12	0.0000
	AE-1	VirB9	870	LC743480	10	0.0000
	AE-1	uvrA	2925	LC743481	40	0.0000
	AE-1	bacA	1248	LC743482	18	0.0000
	AE-1	OMP25	642	LC743483	7	0.0000
	AE-1	OMP10	381	LC743484	4	0.0000
	AE-1	oxyR	954	LC743485	10	0.0000
	AE-1	dnaK	1914	LC743486	25	0.0000
	AE-1	L7-L12	375	LC743487	5	0.0000
	AE-1	OMP19	747	LC743488	10	0.0000
	AE-1	Asp24	381	LC743489	6	0.0000
	AE-1	pifC	405	LC743491	6	0.0000
	AE-1	bvrS	543	LC743492	7	0.0000

**Table 7:** The predicted genes with accession numbers and conserved regions of submitted genes of *B. melitensis* using BIOEDIT

Species	Isolate	Gene	Length (bp)	Accession numbers	Number of conserved regions	Average entropy (Hx)
*B. melitensis*	AE-2	Bp26	753	LC742716	5	0.0000
	AE-2	OMP10	381	LC743471	4	0.0000
	AE-2	DivK	372	LC743472	6	0.0000
	AE-2	rpoB	4134	LC743473	36	0.0000
	AE-2	VirB10	111	LC743493	1	0.0000
	AE-2	nosl	540	LC744194	6	0.0000

#### Molecular Evolutionary

##### Maximum Likelihood Estimation of Substitution Matrix

The Tamura-Nei (1993) model was utilized to calculate substitution patterns and rates, where each element in the model reflects the probability of substitution (r) from one base to another (row to column). Transversional substitution rates were given in *italics*, while transitional substitution rates were shown in **bold**. For the sake of simplicity, the summation of r values is set to 100. A = 21.83%, T/U = 20.96%, C = 28.44%, and G = 28.78% are the nucleotide frequencies (Table S3). To estimate ML values, a tree topology was constructed spontaneously, and the log was -40,491.506. The study involved 30 nucleotide sequences, and gaps as well as incomplete data points were excluded using the full deletion option, resulting in a final dataset with 106 locations (Table S4).

##### Maximum Likelihood Estimation of Transition/Transversion *Bias*

The estimated Transition/Transversion bias (R) is 0.89. Each of the nucleotide frequencies is equal to 25.00%. To estimate ML values, a tree topology was instantly calculated, and the log was − 40,707.647.

##### Average Evolutionary Divergence Over All Sequence Pairs

The rejection odds of the null hypothesis, indicating whether sequences evolved with the same substitution pattern based on differences in base composition biases (Disparity Index test), were determined, and corresponding *P*-values below the diagonal were computed using a Monte Carlo test with 500 replicates. Significant *P*-values are less than 0.05 (highlighted in yellow). An assessment of the disparity index per site is presented for each sequence pair above the diagonal. The study involved 30 nucleotide sequences, with gaps and incomplete data points excluded using the full deletion option, resulting in a dataset of 106 points and an estimated distance of 5.37; the final dataset comprised a total of 4173 locations (Table S5).

#### Phylogenetic Analysis

MEGA11 software was used to analyze the 30 predicted genes using the Maximum Likelihood, Neighbor-joining technique, Minimum-Evolution, Unweighted Pair Group Mean Average “UPGMA” and Maximum Parsimony. Based on the lowest BIC scores (Bayesian Information Criterion), the General Time Reversible model (GTR) was found to be the best model for describing the substitution pattern. Each model’s AICc value (Akaike Information Criterion, corrected), Maximum Likelihood value (lnL), and number of parameters (including branch lengths) are also provided in (Table 8). To account for non-uniformity of evolutionary rates among sites, the discrete Gamma distribution with 5 rate categories (+ G) was used, as well as the assumption that a certain fraction of sites is evolutionarily invariable (+ I). If relevant, gamma shape parameter estimates and/or the fraction of invariant sites are also reported.

**Table 8:** The maximum likelihood fits of 24 different nucleotide substitution models

Model	#Param	BIC	AICc	lnL	Invariant	Gamma	R	Freq A	Freq T	Freq C	Freq G
GTR	65	81,612	81,071	− 40,471	n/a	n/a	0.8755	0.2183	0.2096	0.2844	0.2878
GTR + G	66	81,617	81,069	− 40,468	n/a	26.335	0.9327	0.2183	0.2096	0.2844	0.2878
GTR + I	66	81,622	81,073	− 40,471	0	n/a	0.8759	0.2183	0.2096	0.2844	0.2878
T92	59	81,622	81,132	− 40,507	n/a	n/a	0.8333	0.2139	0.2139	0.2861	0.2861
GTR + G + I	67	81,629	81,072	− 40,469	1E-05	62.722	0.8995	0.2183	0.2096	0.2844	0.2878
T92 + G	60	81,631	81,132	− 40,506	n/a	43.712	0.855	0.2139	0.2139	0.2861	0.2861
T92 + I	60	81,633	81,134	− 40,507	0	n/a	0.8335	0.2139	0.2139	0.2861	0.2861
T92 + G + I	61	81,641	81,134	− 40,506	0	46.219	0.853	0.2139	0.2139	0.2861	0.2861
HKY	61	81,641	81,134	− 40,506	n/a	n/a	0.8348	0.2183	0.2096	0.2844	0.2878
TN93	62	81,644	81,128	− 40,502	n/a	n/a	0.8407	0.2183	0.2096	0.2844	0.2878
HKY + G	62	81,649	81,134	− 40,505	n/a	41.976	0.8576	0.2183	0.2096	0.2844	0.2878
HKY + I	62	81,652	81,136	− 40,506	0	n/a	0.8349	0.2183	0.2096	0.2844	0.2878
TN93 + G	63	81,652	81,128	− 40,501	n/a	44.764	0.862	0.2183	0.2096	0.2844	0.2878
TN93 + I	63	81,654	81,130	− 40,502	1E-05	n/a	0.8408	0.2183	0.2096	0.2844	0.2878
HKY + G + I	63	81,660	81,136	− 40,505	1E-05	44.189	0.8557	0.2183	0.2096	0.2844	0.2878
TN93 + G + I	64	81,662	81,130	− 40,501	1E-05	46.963	0.8603	0.2183	0.2096	0.2844	0.2878
K2	58	82,034	81,551	− 40,718	n/a	n/a	0.887	0.25	0.25	0.25	0.25
K2 + G	59	82,041	81,550	− 40,716	n/a	18.192	0.9265	0.25	0.25	0.25	0.25
K2 + I	59	82,061	81,571	− 40,726	1E-05	n/a	0.9489	0.25	0.25	0.25	0.25
JC	57	82,064	81,590	− 40,738	n/a	n/a	0.5	0.25	0.25	0.25	0.25
JC + G	58	82,073	81,591	− 40,737	n/a	80.18	0.5	0.25	0.25	0.25	0.25
JC + I	58	82,074	81,592	− 40,738	0	n/a	0.5	0.25	0.25	0.25	0.25
K2 + G + I	60	82,078	81,579	− 40,729	1E-05	33.682	1.0135	0.25	0.25	0.25	0.25
JC + G + I	59	82,084	81,593	− 40,737	1E-05	135.15	0.5	0.25	0.25	0.25	0.25

Model	A = > T	A = > C	A = > G	T = > A	T = > C	T = > G	C = > A	C = > T	C = > G	G = > A	G = > T	G = > C
GTR	0.1	0.05	0.12	0.1	0.15	0.04	0.04	0.11	0.09	0.09	0.03	0.08
GTR + G	0.11	0.04	0.13	0.11	0.15	0.04	0.03	0.11	0.09	0.09	0.03	0.08
GTR + I	0.1	0.05	0.12	0.1	0.15	0.04	0.04	0.11	0.09	0.09	0.03	0.08
T92	0.06	0.08	0.13	0.06	0.13	0.08	0.06	0.1	0.08	0.1	0.06	0.08
GTR + G + I	0.1	0.04	0.12	0.11	0.15	0.04	0.03	0.11	0.09	0.09	0.03	0.08
T92 + G	0.06	0.08	0.13	0.06	0.13	0.08	0.06	0.1	0.08	0.1	0.06	0.08
T92 + I	0.06	0.08	0.13	0.06	0.13	0.08	0.06	0.1	0.08	0.1	0.06	0.08
T92 + G + I	0.06	0.08	0.13	0.06	0.13	0.08	0.06	0.1	0.08	0.1	0.06	0.08
HKY	0.06	0.08	0.13	0.06	0.13	0.08	0.06	0.1	0.08	0.1	0.06	0.08
TN93	0.06	0.08	0.12	0.06	0.14	0.08	0.06	0.11	0.08	0.09	0.06	0.08
HKY + G	0.06	0.08	0.13	0.06	0.13	0.08	0.06	0.1	0.08	0.1	0.06	0.08
HKY + I	0.06	0.08	0.13	0.06	0.13	0.08	0.06	0.1	0.08	0.1	0.06	0.08
TN93 + G	0.06	0.08	0.12	0.06	0.15	0.08	0.06	0.11	0.08	0.09	0.06	0.08
TN93 + I	0.06	0.08	0.12	0.06	0.14	0.08	0.06	0.11	0.08	0.09	0.06	0.08
HKY + G + I	0.06	0.08	0.13	0.06	0.13	0.08	0.06	0.1	0.08	0.1	0.06	0.08
TN93 + G + I	0.06	0.08	0.12	0.06	0.15	0.08	0.06	0.11	0.08	0.09	0.06	0.08
K2	0.07	0.07	0.12	0.07	0.12	0.07	0.07	0.12	0.07	0.12	0.07	0.07
K2 + G	0.06	0.06	0.12	0.06	0.12	0.06	0.06	0.12	0.06	0.12	0.06	0.06
K2 + I	0.06	0.06	0.12	0.06	0.12	0.06	0.06	0.12	0.06	0.12	0.06	0.06
JC	0.08	0.08	0.08	0.08	0.08	0.08	0.08	0.08	0.08	0.08	0.08	0.08
JC + G	0.08	0.08	0.08	0.08	0.08	0.08	0.08	0.08	0.08	0.08	0.08	0.08
JC + I	0.08	0.08	0.08	0.08	0.08	0.08	0.08	0.08	0.08	0.08	0.08	0.08
K2 + G + I	0.06	0.06	0.13	0.06	0.13	0.06	0.06	0.13	0.06	0.13	0.06	0.06
JC + G + I	0.08	0.08	0.08	0.08	0.08	0.08	0.08	0.08	0.08	0.08	0.08	0.08

**Fig. 7:**
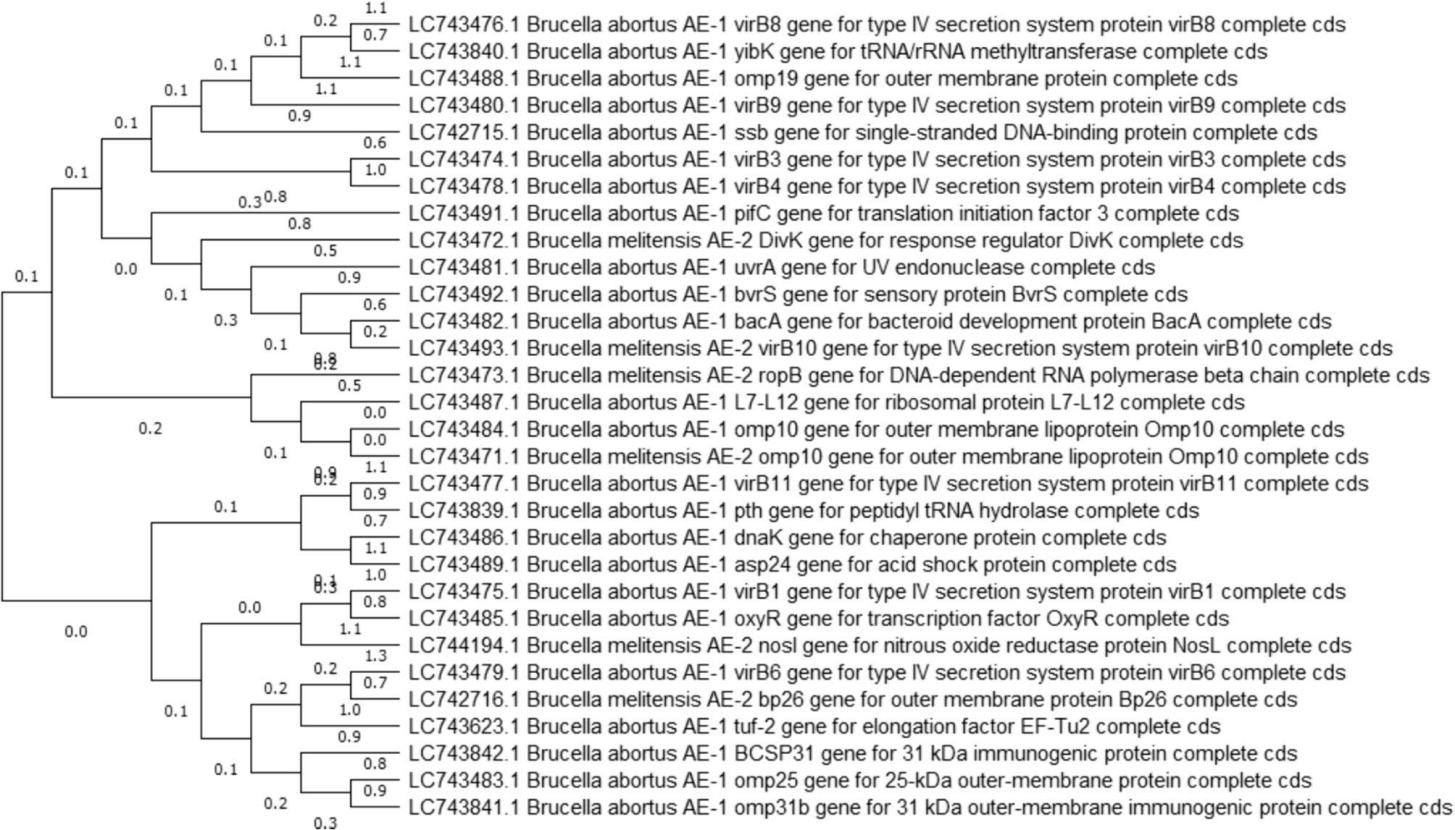
The molecular evolution was assumed using the Maximum Likelihood approach General Time Reversible model. This is the tree with the highest log likelihood (− 40,491.51). Initial tree(s) for the heuristic search were obtained automatically by applying Neighbor-Join and BioNJ algorithms to a matrix of pairwise distances estimated using the Maximum Composite Likelihood (MCL) approach and then selecting the topology with superior log likelihood value. This analysis involved 30 nucleotide sequences. Codon positions included were 1st + 2nd + 3rd + Noncoding. There were a total of 1081 positions in the final dataset

**Fig. 8:**
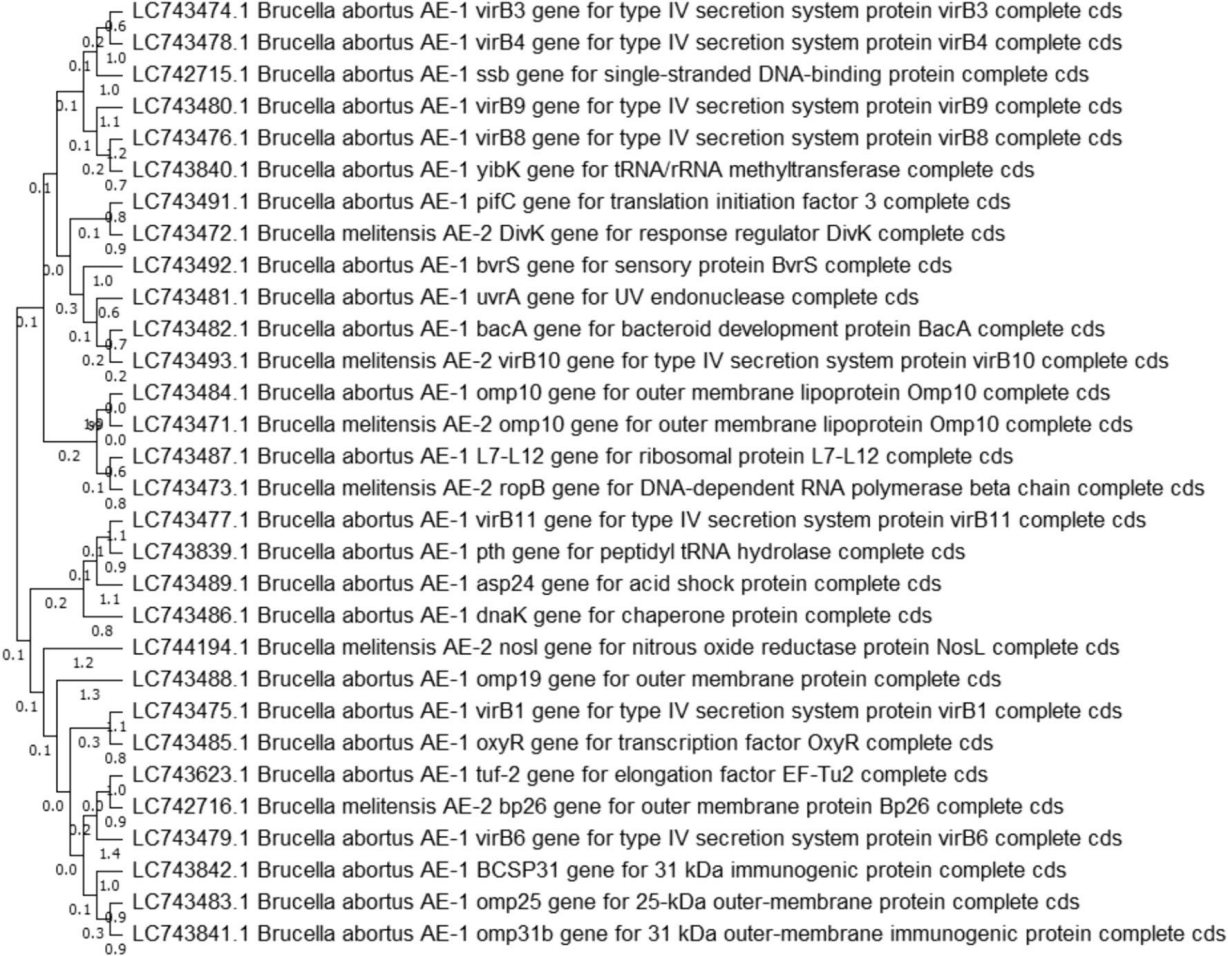
The evolutionary history was inferred by using the Maximum Likelihood method and General Time Reversible model. The tree with the highest log likelihood (− 40,549.08) is shown. The percentage of trees in which the associated taxa clustered together is shown below the branches. Initial tree(s) for the heuristic search were obtained automatically by applying Neighbor-Join and BioNJ algorithms to a matrix of pairwise distances estimated using the Maximum Composite Likelihood (MCL) approach and then selecting the topology with superior log likelihood value. This analysis involved 30 nucleotide sequences. Codon positions included were 1st + 2nd + 3rd + Noncoding. There was a total of 4173 positions in the final dataset

There were no discernible dissimilarities between the two methods. We conducted a phylogenetic analysis with both edited (Fig. 7) and unedited (Fig. 8) alignment and found no distinguishable differences between them. The phylogenetic tree and evolutionary relationships using these approaches were the same for the thirty genes of *B. abortus* and *B. melitensis*, therefore we justified with a maximum likelihood tree (Figs. 7 and 8) and Neighbour-joining tree (Fig. S1). According to the submitted genes, the two *Brucella* species *(abortus* and *melitensis)* were closely related to each other.

## Discussion

Brucellosis is often considered one of the world’s most serious zoonotic diseases. It causes enormous economic losses in both developed and developing countries. Despite massive attempts to eradicate *Brucella* in many countries of the world, this disease has disseminated worldwide (Elrashedy et al. 2022). The complete genome of *Brucella* is 3.3 Mb (approximately 2.1 Mb on chromosome I and 1.2 Mb on chromosome II) (Michaux et al. 1993). Genomic annotation and comparative genome analysis of *Brucella* species are important to reveal the genetic features and get a deep look at the genetic variation in the form of SNPs for validating the vaccine strain. In this study, *B. abortus* strains annotation displayed striking similarities in a variety of features to those previously reported in studies (Wang et al. 2020; Yu et al. 2015). On the other hand, annotation of *B. melitensis* strains was agreed upon in a study performed by (Karthik et al. 2021) and this may be due to the genome stability of *Brucella* this study’s findings confirm that there are significant resemblances between the two *Brucella* species according to the homology degree.

In this study, there are 33 antimicrobial resistance genes identified by the PATRIC server in the two *brucella* species. The intracellular lifestyle of brucellae, which prevents the penetration of certain antimicrobials into the cells, may have a role in the emergence of resistance in these bacteria. As a result, resistance and virulence mechanisms at the genomic, proteomic, and transcriptome levels trigger the alert about an increase in the number of residence genes. The stealthy *Brucella* pathogen has different AMR mechanisms such as enzymatic activation and inactivation of the antimicrobial agent, efflux pumps, replacement of protein or alteration occurring in cell wall charge via protein, drug target modification and resistance genes regulating the gene expression (Biswas et al. 2008; Dadar et al. 2023). DNA gyrase enzyme (gyrA and gyrB) has the primary role in DNA supercoiling, and inhibition of this activity using quinolones or fluoroquinolones generation is combined with rapid killing of the pathogenic *Brucella* (Spencer and Panda 2023). However, rifampicin is composed of four distinguishing subunits (rpoA, rpoB, rpoC and rpoD) and it has a mode of action that inhibits the DNA-dependent RNA polymerase, and its resistance is caused by the mutation at 81 bp in the rpoB gene (Portelli et al. 2020). Also, FosX is an AMR gene that can inactivate Fosfomycin (Bolotin et al. 2021).

Furthermore, efflux pumps can be categorized into six groups, namely, the ATP-binding cassette (ABC), resistance nodulation cell division (RND), major facilitator superfamily (MFS), multi-antimicrobial and toxic compound extrusion (MATE), proteobacterial antimicrobial compound efflux (PACE) and small multidrug resistance (SMR) family (Du et al. 2018). In this study, efflux pumps in two families (ABC and RND) were accompanied by antibiotic resistance. MacAB (macrolide-specific ABC-type efflux carrier) involves macA and macB genes that perform resistance against macrolides composed of 14- and 15-membered compounds (Greene et al. 2018). The RND family is another efflux pump that features the triclosan-specific efflux protein TriABC-OpmH. In *Pseudomonas aeruginosa*, this protein comprises two membrane fusion proteins, TriA and TriB (Fabre et al. 2021). Although its role in *Brucella* is not yet understood, increased resistance to ciprofloxacin and imipenem suggests that the efflux pump in *Brucella* may provide quinolone resistance.

What’s more, folic acid (vitamin B9) is an important amino acid that enters the folate synthesis process which is vital for the replication of DNA. This process can be blocked by trimethoprim and sulfamethoxazole. The presence or absence of the folA (dihydrofolate reductase) or the folP (dihydropteroate synthase) can produce resistance to trimethoprim and sulfamethoxazole respectively (Biswas et al. 2008; Nurjadi et al. 2021). Additionally, gidB gene encodes a tRNA uridine 5-carboxymethyl aminomethyl modification enzyme that participates in post-transcriptional tRNA modifications. The gidB exhibited a decreased susceptibility to aminoglycoside antibiotics like gentamicin and streptomycin (Mikheil et al. 2012).

In *Brucella*, OxyR is a transcription factor that affects the bacteria’s ability to combat oxidative stress and resist reactive oxygen species (ROS) produced within the host cell. This protein controls the expression of different cellular components responsible for antioxidant defense mechanisms. It can trigger the activation of numerous genes, including catalase-peroxidase, superoxide dismutase, and other detoxification genes, which aid in the bacteria’s protection against ROS-induced damage (Kim and Mayfield 2000; Wang et al. 2022). Moreover, PgsA and GdpD contribute to the lipopolysaccharide (LPS) that has a very important role in *Brucella* virulence and survival (Elrashedy et al. 2022). In addition, these genes Alr, Ddl, S10p, S12p, EF-G, inhA, EF-Tu, Iso-tRNA, Dfr, kasA, rho, MurA, and fabI have been implicated in various cellular functions and pathways in *Brucella*. While some of these genes have been implicated in antibiotic resistance in other bacteria, their role in *Brucella* antibiotic resistance may not be extensively studied or well-established.

In this study, there are some differences in Fayioum strains of *B. meliteinsis* revealed in antimicrobial resistance genes which can be explained on the basis that Fayioum main raising cattle is beef collected from different sources (local, European, and Latin) with no vaccination representing a pool for mixing different *Brucella* strains from different sources. KatG is a gene that encodes for the catalase-peroxidase enzyme. This enzyme is responsible for the detoxification of ROS within bacterial cells and is therefore considered to play a key role in bacterial defense mechanisms, including resistance to antibiotics. Although KatG is not typically considered as a primary antibiotic resistance gene in *Brucella*, studies have shown that mutations in this gene can result in resistance to isoniazid, an anti-tuberculosis drug that is also used to treat brucellosis (Bollela et al. 2016). Additionally, The Erm(C) gene is a ribosomal RNA methylase gene that confers resistance to macrolide-lacosamide-streptogramin B (MLSB) antibiotics, including erythromycin, clindamycin, and streptogramin B (Khodabandeh et al. 2019). From the above mentioned, antibiotic-resistance genes have the potential to spread between different bacterial species through horizontal gene transfer, leading to the emergence of multidrug-resistant strains that are difficult to treat. Hence, it is crucial to monitor antibiotic resistance patterns across various bacterial species and develop suitable treatment strategies to ensure the effective management of bacterial infections.

In this study, whole genome phylogeny analysis for all *Brucella* strains was classified into six clades: *B. melitensis* clade; *B. abortus* clade; *B. canis clade*; *B. suis clade; B. microti*; *B. pinnipedialis*; *B. ceti* clade; and *B. ovis* clade. The evolutionary relationship constructed here fits with previous results (Azam et al. 2016). The transition mutations occur more frequently than transversion mutations and could have an impact on the evolution of protein-coding genes. This is because the DNA sequence undergoes less significant changes during transition mutations, which may result in fewer negative consequences on the protein-coding sequence. As a result, certain parts of the genome could experience higher rates of transition substitutions, possibly due to the less deleterious impact of these mutations on protein function in those regions.

By analyzing the pan-genome, we can gain a comprehensive understanding of the gene content and genetic evolution within a population, which provides valuable insights. Additionally, identifying core and accessory genes through pan-genome analysis can help us to better comprehend the genetic relatedness and variability of different strains of a particular species. When we apply this mechanism to *B. abortus* and *B. melitensis*, it suggests that these two species have a significant genetic resemblance, which is also supported by the absence of accessory genes in the sampled strains. In this study, the results are in agreement with (Wattam et al. 2014) who reported 2285 core genes in the *Brucella* species. Finally, This study enhances our knowledge of the epidemiology and evolution of brucellosis in Egypt by providing significant insights into the genetic diversity, potential virulence factors, and associations related to vaccines of *Brucella* pathogens.

## Conclusion

Our investigation into Brucellosis involves the genomic analysis of eight *B. abortus* and eighteen *B. melitensis* strains from Egypt have yielded significant findings. The comprehensive comparison with RB51 and REV1 vaccines, annotation of genome characteristics, identification of antimicrobial resistance genes (AMR), and elucidation of phylogenetic relationships offer an understanding of the genetic landscape of this *Brucella* pathogen. The detection of AMR in two *Brucella* species indicates that Brucellae possess several strategies to withstand antimicrobial agents. One of these strategies is the intracellular lifestyle of *Brucella*, which hinders the entry of specific antimicrobials into the cells, contributing to the development of resistance. Also in this study, we predicted and submitted thirty candidate genes to GenBank. Interestingly, the distinct features observed in *B. melitensis* strains, particularly in the Faiyum isolate, highlight potential variations in virulence and resistance profiles. The clustering patterns in the whole genome phylogeny further emphasize the geographical relevance, linking *B. abortus* strains to vaccinated animals and grouping *B. melitensis* strains from Menoufia, Gharbia, Dameitta, and Kafr Elshiek. The detection of numerous SNPs and subsequent annotation enhances our knowledge of the molecular intricacies of the *Brucella* pathogen. This research increases our understanding of the epidemiology and evolution of brucellosis in Egypt by providing useful insights into the genetic diversity, putative virulence factors, and vaccine-related associations of these *Brucella* pathogens.

### Supplementary Information

Below is the link to the electronic supplementary material.

Supplementary file1 (DOCX 122 KB)

## Data Availability

All the primary data used in the study were included in the article (and its supplementary files).
