# Supplementary material for: Phylogenetic Analysis and Comparative Genomics of Brucella abortus and Brucella melitensis Strains in Egypt
Source: J Mol Evol. 2024 May 29;92(3):338–57. doi: 10.1007/s00239-024-10173-0 (PMC11169049; doi:10.1007/s00239-024-10173-0)
Supplement: Supplementary file 1 — Supplementary file1 (DOCX 122 KB) [file 239_2024_10173_MOESM1_ESM.docx]

**Phylogenetic Analysis and Comparative Genomics of *Brucella abortus* and *Brucella melitensis* Strains in Egypt**

**Alyaa Elrashedy^1*^; Mohamed Nayel^1^; Akram Salama^1^; Ahmed Zaghawa^1^ , Nader R. Abdelsalam^2^** & **Mohamed E. Hasan^3^**

^1^ Department of Animal Medicine and Infectious Diseases (Infectious Diseases), Faculty of Veterinary Medicine, University of Sadat City, Egypt.

^2^Agricultural Botany Department, Faculty of Agriculture (Saba Basha), Alexandria University, 21531, Alexandria, Egypt

^3^Bioinformatics Department, Genetic Engineering and Biotechnology Research Institute, University of Sadat City, Egypt.

* Corresponding author: [Alyaa.elrashedy.ms@vet.usc.edu.eg](mailto:Alyaa.elrashedy.ms@vet.usc.edu.eg)

[alyaaelrshedy96@gmail.com](mailto:alyaaelrshedy96@gmail.com)

**Table (S1): List of Egyptian *Brucella abortus* strains used in this study**

| **Organism** | **Accession numbers** | **Biosample** | **Bioproject** | **Country** | **Host** | **Isolation source** | **Year** | **Reference** |
| --- | --- | --- | --- | --- | --- | --- | --- | --- |
| *Brucella abortus* | [SRR12368029](https://trace.ncbi.nlm.nih.gov/Traces/sra?run=SRR12368029) | [SAMN15701920](https://www.ncbi.nlm.nih.gov/biosample/SAMN15701920) | PRJNA650270 | Egypt: Giza, Al-Badrashein | Cattle | lymph node | 2017 | (Khan et al. 2021) |
|  | [SRR12368032](https://trace.ncbi.nlm.nih.gov/Traces/sra?run=SRR12368032) | [SAMN15701943](https://www.ncbi.nlm.nih.gov/biosample/SAMN15701943) | PRJNA650270 | Egypt: Qalyubia, Toukh | Cattle | lymph node | 2017 |  |
|  | [SRR12368034](https://trace.ncbi.nlm.nih.gov/Traces/sra?run=SRR12368034) | [SAMN15701941](https://www.ncbi.nlm.nih.gov/biosample/SAMN15701941) | PRJNA650270 | Egypt: Monufia, Sirs Al-Layyan | Cattle | lymph node | 2017 |  |
|  | [SRR12368035](https://trace.ncbi.nlm.nih.gov/Traces/sra?run=SRR12368035) | [SAMN15701940](https://www.ncbi.nlm.nih.gov/biosample/SAMN15701940) | PRJNA650270 | Egypt: Dakahlia, Mansoura | Cattle | lymph node | 2017 |  |
|  | [SRR12368036](https://trace.ncbi.nlm.nih.gov/Traces/sra?run=SRR12368036) | [SAMN15701939](https://www.ncbi.nlm.nih.gov/biosample/SAMN15701939) | PRJNA650270 | Egypt: Beheira, Ad-Dilinjat | Cattle | Fetal stomach content | 2017 |  |
|  | [SRR12368047](https://trace.ncbi.nlm.nih.gov/Traces/sra?run=SRR12368047) | [SAMN15701929](https://www.ncbi.nlm.nih.gov/biosample/SAMN15701929) | PRJNA650270 | Egypt: Asyut, Asyut | Buffalo | lymph node | 2017 |  |
|  | [SRR12368049](https://trace.ncbi.nlm.nih.gov/Traces/sra?run=SRR12368049) | [SAMN15701927](https://www.ncbi.nlm.nih.gov/biosample/SAMN15701927) | PRJNA650270 | Egypt: Beheira, Damanhour | Cattle | lymph node | 2016 |  |
|  | [SRR12368050](https://trace.ncbi.nlm.nih.gov/Traces/sra?run=SRR12368050) | [SAMN15701926](https://www.ncbi.nlm.nih.gov/biosample/SAMN15701926) | PRJNA650270 | Egypt: Beheira, Damanhour | Cattle | lymph node | 2016 |  |

**Table (S2) List of Egyptian *Brucella melitensis* strains**

| **Organism** | **Accession numbers** | **Biosample** | **Bioproject** | **Country** | **Host** | **Isolation source** | **Year** | **Reference** |
| --- | --- | --- | --- | --- | --- | --- | --- | --- |
| *Bricella melitensis* | [SRR19520306](https://trace.ncbi.nlm.nih.gov/Traces/sra?run=SRR19520306) | [SAMN28827632](https://www.ncbi.nlm.nih.gov/biosample/SAMN28827632) | [PRJNA773199](https://www.ncbi.nlm.nih.gov/bioproject/PRJNA773199) | Egypt: Monufia, Ashmoun | ewe |  | 2013 | (Holzer et al. 2022) |
|  | [SRR19520359](https://trace.ncbi.nlm.nih.gov/Traces/sra?run=SRR19520359) | [SAMN28827584](https://www.ncbi.nlm.nih.gov/biosample/SAMN28827584) | [PRJNA773199](https://www.ncbi.nlm.nih.gov/bioproject/PRJNA773199) | Egypt: Monufia | goat |  | 2017 |  |
|  | [SRR19520360](https://trace.ncbi.nlm.nih.gov/Traces/sra?run=SRR19520360) | [SAMN28827583](https://www.ncbi.nlm.nih.gov/biosample/SAMN28827583) | [PRJNA773199](https://www.ncbi.nlm.nih.gov/bioproject/PRJNA773199) | Egypt: Monufia | goat |  | 2017 |  |
|  | [SRR19520406](https://trace.ncbi.nlm.nih.gov/Traces/sra?run=SRR19520406) | [SAMN28827664](https://www.ncbi.nlm.nih.gov/biosample/SAMN28827664) | [PRJNA773199](https://www.ncbi.nlm.nih.gov/bioproject/PRJNA773199) | Egypt: Monufia, Ashmoun | buffalo |  | 2019 |  |
|  | [SRR19520407](https://trace.ncbi.nlm.nih.gov/Traces/sra?run=SRR19520407) | [SAMN28827663](https://www.ncbi.nlm.nih.gov/biosample/SAMN28827663) | [PRJNA773199](https://www.ncbi.nlm.nih.gov/bioproject/PRJNA773199) | Egypt: Monufia, Ashmoun | cattle |  | 2019 |  |
|  | [SRR19520408](https://trace.ncbi.nlm.nih.gov/Traces/sra?run=SRR19520408) | [SAMN28827662](https://www.ncbi.nlm.nih.gov/biosample/SAMN28827662) | [PRJNA773199](https://www.ncbi.nlm.nih.gov/bioproject/PRJNA773199) | Egypt: Monufia, Ashmoun | cattle |  | 2019 |  |
|  | [SRR19520400](https://trace.ncbi.nlm.nih.gov/Traces/sra?run=SRR19520400) | [SAMN28827670](https://www.ncbi.nlm.nih.gov/biosample/SAMN28827670) | [PRJNA773199](https://www.ncbi.nlm.nih.gov/bioproject/PRJNA773199) | Egypt: Monufia, Ashmoun | cattle |  | 2019 |  |
|  | [SRR19520401](https://trace.ncbi.nlm.nih.gov/Traces/sra?run=SRR19520401) | [SAMN28827669](https://www.ncbi.nlm.nih.gov/biosample/SAMN28827669) | [PRJNA773199](https://www.ncbi.nlm.nih.gov/bioproject/PRJNA773199) | Egypt: Monufia, Ashmoun | cattle |  | 2019 |  |
|  | [SRR19520402](https://trace.ncbi.nlm.nih.gov/Traces/sra?run=SRR19520402) | [SAMN28827668](https://www.ncbi.nlm.nih.gov/biosample/SAMN28827668) | [PRJNA773199](https://www.ncbi.nlm.nih.gov/bioproject/PRJNA773199) | Egypt: Monufia, Ashmoun | cattle |  | 2019 |  |
|  | [SRR19520403](https://trace.ncbi.nlm.nih.gov/Traces/sra?run=SRR19520403) | [SAMN28827667](https://www.ncbi.nlm.nih.gov/biosample/SAMN28827667) | [PRJNA773199](https://www.ncbi.nlm.nih.gov/bioproject/PRJNA773199) | Egypt: Monufia, Shebeen El-Kom | buffalo |  | 2019 |  |
|  | [SRR19520405](https://trace.ncbi.nlm.nih.gov/Traces/sra?run=SRR19520405) | [SAMN28827665](https://www.ncbi.nlm.nih.gov/biosample/SAMN28827665) | [PRJNA773199](https://www.ncbi.nlm.nih.gov/bioproject/PRJNA773199) | Egypt: Monufia, Ashmoun | buffalo |  | 2019 |  |
|  | [SRR19520399](https://trace.ncbi.nlm.nih.gov/Traces/sra?run=SRR19520399) | [SAMN28827671](https://www.ncbi.nlm.nih.gov/biosample/SAMN28827671) | [PRJNA773199](https://www.ncbi.nlm.nih.gov/bioproject/PRJNA773199) | Egypt: Monufia, Birket El Sab | cattle |  | 2019 |  |
|  | [SRR19520404](https://trace.ncbi.nlm.nih.gov/Traces/sra?run=SRR19520404) | [SAMN28827666](https://www.ncbi.nlm.nih.gov/biosample/SAMN28827666) | [PRJNA773199](https://www.ncbi.nlm.nih.gov/bioproject/PRJNA773199) | Egypt: Monufia, Birket El Sab | cattle |  | 2018 |  |
|  | [SRR19520392](https://trace.ncbi.nlm.nih.gov/Traces/sra?run=SRR19520392) | [SAMN28827677](https://www.ncbi.nlm.nih.gov/biosample/SAMN28827677) | [PRJNA773199](https://www.ncbi.nlm.nih.gov/bioproject/PRJNA773199) | Egypt: Monufia, Menouf | buffalo |  | 2018 |  |
|  | [SRR19520393](https://trace.ncbi.nlm.nih.gov/Traces/sra?run=SRR19520393) | [SAMN28827676](https://www.ncbi.nlm.nih.gov/biosample/SAMN28827676) | [PRJNA773199](https://www.ncbi.nlm.nih.gov/bioproject/PRJNA773199) | Egypt: Monufia, Menouf | cattle |  | 2018 |  |
|  | [SRR19520394](https://trace.ncbi.nlm.nih.gov/Traces/sra?run=SRR19520394) | [SAMN28827675](https://www.ncbi.nlm.nih.gov/biosample/SAMN28827675) | [PRJNA773199](https://www.ncbi.nlm.nih.gov/bioproject/PRJNA773199) | Egypt: Monufia, Shuhada | cattle |  | 2018 |  |
|  | [SRR19520395](https://trace.ncbi.nlm.nih.gov/Traces/sra?run=SRR19520395) | [SAMN28827674](https://www.ncbi.nlm.nih.gov/biosample/SAMN28827674) | [PRJNA773199](https://www.ncbi.nlm.nih.gov/bioproject/PRJNA773199) | Egypt: Monufia, Shuhada | cattle |  | 2018 |  |
|  | [SRR19520396](https://trace.ncbi.nlm.nih.gov/Traces/sra?run=SRR19520396) | [SAMN28827673](https://www.ncbi.nlm.nih.gov/biosample/SAMN28827673) | [PRJNA773199](https://www.ncbi.nlm.nih.gov/bioproject/PRJNA773199) | Egypt: Monufia, Shuhada | cattle |  | 2018 |  |
|  | [SRR19520411](https://trace.ncbi.nlm.nih.gov/Traces/sra?run=SRR19520411) | [SAMN28827660](https://www.ncbi.nlm.nih.gov/biosample/SAMN28827660) | [PRJNA773199](https://www.ncbi.nlm.nih.gov/bioproject/PRJNA773199) | Egypt: Monufia, Sherbeen El-Kom | buffalo |  | 2019 |  |
|  | [SRR19520397](https://trace.ncbi.nlm.nih.gov/Traces/sra?run=SRR19520397) | [SAMN28827672](https://www.ncbi.nlm.nih.gov/biosample/SAMN28827672) | [PRJNA773199](https://www.ncbi.nlm.nih.gov/bioproject/PRJNA773199) | Egypt: Monufia, Shuhada | cattle |  | 2018 |  |
|  | [SRR19520319](https://trace.ncbi.nlm.nih.gov/Traces/sra?run=SRR19520319) | [SAMN28827621](https://www.ncbi.nlm.nih.gov/biosample/SAMN28827621) | [PRJNA773199](https://www.ncbi.nlm.nih.gov/bioproject/PRJNA773199) | Egypt: Faiyum, Tamiya | cow |  | 2007 |  |
|  | [SRR19520422](https://trace.ncbi.nlm.nih.gov/Traces/sra?run=SRR19520422) | [SAMN28827650](https://www.ncbi.nlm.nih.gov/biosample/SAMN28827650) | [PRJNA773199](https://www.ncbi.nlm.nih.gov/bioproject/PRJNA773199) | Egypt: Faiyum, Dimu | cow |  | 2014 |  |
|  | [SRR19520320](https://trace.ncbi.nlm.nih.gov/Traces/sra?run=SRR19520320) | [SAMN28827620](https://www.ncbi.nlm.nih.gov/biosample/SAMN28827620) | [PRJNA773199](https://www.ncbi.nlm.nih.gov/bioproject/PRJNA773199) | Egypt: Sharqia, Minya Al-Qamh | ewe |  | 2007 |  |
|  | [SRR19520326](https://trace.ncbi.nlm.nih.gov/Traces/sra?run=SRR19520326) | [SAMN28827614](https://www.ncbi.nlm.nih.gov/biosample/SAMN28827614) | [PRJNA773199](https://www.ncbi.nlm.nih.gov/bioproject/PRJNA773199) | Egypt: Matruh, Al-Dabaa | goat |  | 2015 |  |
|  | [SRR19520361](https://trace.ncbi.nlm.nih.gov/Traces/sra?run=SRR19520361) | [SAMN28827582](https://www.ncbi.nlm.nih.gov/biosample/SAMN28827582) | [PRJNA773199](https://www.ncbi.nlm.nih.gov/bioproject/PRJNA773199) | Egypt: Gharbia | sheep |  | 2017 |  |
|  | [SRR19520382](https://trace.ncbi.nlm.nih.gov/Traces/sra?run=SRR19520382) | [SAMN28827563](https://www.ncbi.nlm.nih.gov/biosample/SAMN28827563) | [PRJNA773199](https://www.ncbi.nlm.nih.gov/bioproject/PRJNA773199) | Egypt: Kafr El Sheik | cattle |  | 2017 |  |
|  | [SRR19520411](https://trace.ncbi.nlm.nih.gov/Traces/sra?run=SRR19520411) | [SAMN28827660](https://www.ncbi.nlm.nih.gov/biosample/SAMN28827660) | [PRJNA773199](https://www.ncbi.nlm.nih.gov/bioproject/PRJNA773199) | Egypt: Monufia, Sherbeen El-Kom | buffalo |  | 2019 |  |
|  | [SRR19520420](https://trace.ncbi.nlm.nih.gov/Traces/sra?run=SRR19520420) | [SAMN28827553](https://www.ncbi.nlm.nih.gov/biosample/SAMN28827553) | [PRJNA773199](https://www.ncbi.nlm.nih.gov/bioproject/PRJNA773199) | Egypt: Damietta | camel |  | 2015 |  |
|  | [SRR19520421](https://trace.ncbi.nlm.nih.gov/Traces/sra?run=SRR19520421) | [SAMN28827651](https://www.ncbi.nlm.nih.gov/biosample/SAMN28827651) | [PRJNA773199](https://www.ncbi.nlm.nih.gov/bioproject/PRJNA773199) | Egypt: Dakahlia, Somosta | buffalo |  | 2014 |  |
|  | [SRR19520334](https://trace.ncbi.nlm.nih.gov/Traces/sra?run=SRR19520334) | [SAMN28827607](https://www.ncbi.nlm.nih.gov/biosample/SAMN28827607) | [PRJNA773199](https://www.ncbi.nlm.nih.gov/bioproject/PRJNA773199) | Egypt: Aswan | sheep |  | 2019 |  |
|  | [SRR12368024](https://trace.ncbi.nlm.nih.gov/Traces/sra?run=SRR12368024) | [SAMN15701925](https://www.ncbi.nlm.nih.gov/biosample/SAMN15701925) | [PRJNA650270](https://www.ncbi.nlm.nih.gov/bioproject/PRJNA650270) | Egypt: Beheira, Damanhour | Cattle | lymph node | 2016 | (Khan et al. 2021) |
|  | [SRR12368025](https://trace.ncbi.nlm.nih.gov/Traces/sra?run=SRR12368025) | [SAMN15701924](https://www.ncbi.nlm.nih.gov/biosample/SAMN15701924) | [PRJNA650270](https://www.ncbi.nlm.nih.gov/bioproject/PRJNA650270) | Egypt: Beheira, Damanhour | Cattle | lymph node | 2016 |  |
|  | [SRR12368031](https://trace.ncbi.nlm.nih.gov/Traces/sra?run=SRR12368031) | [SAMN15701944](https://www.ncbi.nlm.nih.gov/biosample/SAMN15701944) | PRJNA650270 | Egypt: Qalyubia, Toukh | Buffalo | lymph node | 2017 |  |
|  | [SRR12368033](https://trace.ncbi.nlm.nih.gov/Traces/sra?run=SRR12368033) | [SAMN15701942](https://www.ncbi.nlm.nih.gov/biosample/SAMN15701942) | PRJNA650270 | Egypt: Monufia, Sirs Al-Layyan | Cattle | milk | 2017 |  |
|  | [SRR12368037](https://trace.ncbi.nlm.nih.gov/Traces/sra?run=SRR12368037) | [SAMN15701938](https://www.ncbi.nlm.nih.gov/biosample/SAMN15701938) | PRJNA650270 | Egypt: Ismailia, Ismailia | Cattle | lymph node | 2017 |  |
|  | [SRR12368040](https://trace.ncbi.nlm.nih.gov/Traces/sra?run=SRR12368040) | [SAMN15701918](https://www.ncbi.nlm.nih.gov/biosample/SAMN15701918) | PRJNA650270 | Egypt: Giza, Al-Badrashein | Cattle | lymph node | 2017 |  |
|  | [SRR12368042](https://trace.ncbi.nlm.nih.gov/Traces/sra?run=SRR12368042) | [SAMN15701934](https://www.ncbi.nlm.nih.gov/biosample/SAMN15701934) | PRJNA650270 | Egypt: Beni Suef, Al-Wasta | Sheep | lymph node | 2015 |  |
|  | [SRR12368048](https://trace.ncbi.nlm.nih.gov/Traces/sra?run=SRR12368048) | [SAMN15701928](https://www.ncbi.nlm.nih.gov/biosample/SAMN15701928) | PRJNA650270 | Egypt: Asyut, Asyut | Buffalo | lymph node | 2017 |  |

**Table (S3) Maximum Likelihood Estimation of Substitution Matrix**

| From\To | A | T | C | G |
| --- | --- | --- | --- | --- |
| A | - | *5.6148* | *7.6166* | **11.9023** |
| T | *5.8460* | - | **14.6790** | *7.7078* |
| C | *5.8460* | **10.8211** | - | *7.7078* |
| G | **9.0273** | *5.6148* | *7.6166* | - |

**Table (S4) Maximum Composite Likelihood Estimation Pattern of Nucleotide Substitution**

|  | **A** | **T** | **C** | **G** |
| --- | --- | --- | --- | --- |
| **A** | - | *1.92* | *2.67* | **5.73** |
| **T** | *1.83* | - | **41.95** | *2.73* |
| **C** | *1.83* | **30.17** | - | *2.73* |
| **G** | **3.85** | *1.92* | *2.67* | - |

**Table (S5) Test of the Homogeneity of Substitution Patterns Between Sequences**

|  | 1 | 2 | 3 | 4 | 5 | 6 | 7 | 8 | 9 | 10 | 11 | 12 | 13 | 14 | 15 | 16 | 17 | 18 | 19 | 20 | 21 | 22 | 23 | 24 | 25 | 26 | 27 | 28 | 29 | 30 |
| --- | --- | --- | --- | --- | --- | --- | --- | --- | --- | --- | --- | --- | --- | --- | --- | --- | --- | --- | --- | --- | --- | --- | --- | --- | --- | --- | --- | --- | --- | --- |
| LC743474.1 |  | 0.00 | 0.55 | 0.17 | 0.00 | 0.00 | 0.00 | 0.34 | 0.00 | 0.18 | 0.00 | 0.00 | 0.31 | 0.00 | 0.03 | 0.46 | 0.00 | 0.10 | 2.19 | 0.63 | 0.00 | 0.00 | 0.72 | 0.32 | 0.00 | 0.00 | 0.32 | 1.11 | 4.15 | 1.15 |
| LC743475.1 | 1.00 |  | 0.00 | 0.00 | 0.00 | 1.10 | 0.00 | 0.00 | 1.00 | 0.00 | 0.24 | 0.12 | 0.17 | 0.00 | 0.63 | 0.00 | 0.11 | 1.79 | 1.12 | 0.61 | 0.00 | 0.00 | 0.97 | 0.21 | 0.00 | 0.17 | 0.32 | 0.16 | 3.36 | 0.00 |
| LC743476.1 | 0.14 | 1.00 |  | 0.31 | 0.00 | 2.70 | 0.09 | 0.00 | 2.88 | 0.37 | 0.91 | 1.07 | 0.70 | 0.20 | 1.73 | 0.00 | 1.27 | 2.76 | 0.28 | 1.43 | 0.71 | 0.00 | 2.26 | 1.28 | 0.00 | 0.84 | 0.00 | 1.27 | 4.14 | 0.00 |
| LC743477.1 | 0.25 | 1.00 | 0.23 |  | 0.25 | 1.37 | 0.00 | 0.00 | 2.92 | 0.00 | 0.60 | 0.26 | 0.00 | 0.00 | 0.00 | 0.00 | 0.30 | 2.16 | 0.60 | 0.53 | 0.00 | 0.00 | 0.55 | 1.05 | 0.54 | 0.56 | 0.54 | 0.00 | 2.34 | 0.00 |
| LC743478.1 | 1.00 | 1.00 | 1.00 | 0.27 |  | 1.57 | 0.00 | 0.00 | 6.03 | 0.27 | 0.27 | 0.00 | 0.33 | 0.62 | 1.12 | 0.29 | 0.00 | 1.21 | 1.81 | 1.05 | 0.39 | 0.00 | 4.02 | 0.25 | 0.00 | 0.20 | 0.81 | 0.70 | 3.50 | 0.01 |
| LC743479.1 | 1.00 | 0.06 | 0.00 | 0.04 | 0.03 |  | 0.68 | 1.22 | 0.00 | 0.20 | 0.52 | 0.64 | 3.59 | 1.03 | 0.58 | 1.12 | 0.30 | 0.00 | 4.73 | 0.98 | 1.17 | 1.78 | 0.47 | 0.00 | 1.49 | 0.41 | 1.43 | 0.00 | 3.91 | 0.90 |
| LC743480.1 | 1.00 | 1.00 | 0.34 | 1.00 | 1.00 | 0.14 |  | 0.00 | 0.78 | 0.00 | 0.00 | 0.00 | 0.23 | 0.00 | 0.08 | 0.00 | 0.00 | 2.03 | 1.51 | 1.01 | 0.32 | 0.00 | 1.56 | 0.02 | 0.00 | 0.00 | 0.62 | 0.64 | 6.82 | 0.00 |
| LC742715.1 | 0.21 | 1.00 | 1.00 | 1.00 | 1.00 | 0.07 | 1.00 |  | 0.93 | 0.00 | 1.37 | 0.65 | 0.18 | 0.00 | 0.00 | 0.00 | 0.18 | 2.44 | 0.72 | 0.81 | 0.00 | 0.00 | 0.00 | 0.02 | 0.00 | 1.30 | 0.35 | 0.00 | 6.06 | 0.00 |
| LC743482.1 | 1.00 | 0.06 | 0.00 | 0.00 | 0.00 | 1.00 | 0.10 | 0.08 |  | 0.53 | 1.33 | 0.79 | 7.29 | 3.52 | 0.63 | 1.39 | 0.40 | 0.00 | 4.97 | 0.62 | 2.25 | 1.92 | 0.98 | 0.05 | 1.68 | 1.20 | 1.65 | 2.14 | 1.56 | 0.70 |
| LC743483.1 | 0.28 | 1.00 | 0.23 | 1.00 | 0.24 | 0.28 | 1.00 | 1.00 | 0.15 |  | 1.48 | 0.60 | 0.12 | 0.00 | 0.00 | 0.00 | 0.12 | 1.60 | 0.78 | 0.00 | 0.00 | 0.00 | 0.00 | 0.00 | 0.00 | 1.39 | 0.00 | 0.00 | 2.47 | 0.00 |
| LC743484.1 | 1.00 | 0.24 | 0.08 | 0.15 | 0.24 | 0.17 | 1.00 | 0.03 | 0.01 | 0.02 |  | 0.00 | 1.04 | 0.07 | 1.56 | 0.92 | 1.03 | 1.61 | 3.50 | 3.10 | 1.25 | 0.00 | 1.46 | 2.31 | 1.15 | 0.00 | 3.25 | 2.60 | 5.59 | 2.59 |
| LC743485.1 | 1.00 | 0.30 | 0.06 | 0.25 | 1.00 | 0.10 | 1.00 | 0.12 | 0.12 | 0.14 | 1.00 |  | 2.35 | 0.31 | 1.96 | 1.29 | 0.00 | 0.75 | 4.26 | 1.33 | 0.84 | 0.31 | 2.34 | 1.23 | 0.00 | 0.00 | 2.86 | 0.92 | 5.33 | 0.61 |
| LC743486.1 | 0.26 | 0.33 | 0.09 | 1.00 | 0.23 | 0.00 | 0.26 | 0.30 | 0.00 | 0.31 | 0.06 | 0.00 |  | 0.47 | 0.66 | 0.00 | 1.02 | 1.49 | 2.14 | 0.32 | 0.00 | 0.12 | 4.88 | 2.37 | 0.64 | 0.98 | 1.12 | 0.90 | 1.45 | 0.00 |
| LC743481.1 | 1.00 | 1.00 | 0.26 | 1.00 | 0.14 | 0.06 | 1.00 | 1.00 | 0.00 | 1.00 | 0.36 | 0.23 | 0.15 |  | 0.42 | 0.00 | 0.00 | 0.71 | 2.44 | 0.96 | 0.00 | 0.00 | 2.58 | 0.30 | 0.00 | 0.01 | 0.94 | 0.02 | 5.48 | 0.00 |
| LC743842.1 | 0.36 | 0.12 | 0.01 | 1.00 | 0.06 | 0.15 | 0.39 | 1.00 | 0.13 | 1.00 | 0.01 | 0.01 | 0.09 | 0.18 |  | 0.00 | 1.57 | 1.25 | 1.53 | 0.00 | 0.16 | 1.07 | 0.00 | 0.24 | 1.20 | 1.46 | 0.19 | 0.34 | 3.18 | 0.00 |
| LC743487.1 | 0.19 | 1.00 | 1.00 | 1.00 | 0.24 | 0.05 | 1.00 | 1.00 | 0.03 | 1.00 | 0.05 | 0.04 | 1.00 | 1.00 | 1.00 |  | 0.42 | 0.75 | 0.62 | 0.37 | 0.00 | 0.00 | 0.00 | 0.00 | 0.00 | 0.85 | 0.18 | 0.11 | 2.22 | 0.00 |
| LC743488.1 | 1.00 | 0.32 | 0.03 | 0.22 | 1.00 | 0.25 | 1.00 | 0.30 | 0.19 | 0.33 | 0.07 | 1.00 | 0.06 | 1.00 | 0.02 | 0.15 |  | 0.37 | 2.71 | 1.34 | 0.30 | 0.10 | 0.79 | 0.64 | 0.09 | 0.90 | 0.48 | 0.60 | 6.14 | 0.81 |
| LC743489.1 | 0.33 | 0.01 | 0.00 | 0.00 | 0.04 | 1.00 | 0.01 | 0.00 | 1.00 | 0.01 | 0.03 | 0.10 | 0.02 | 0.15 | 0.06 | 0.10 | 0.21 |  | 5.68 | 0.28 | 0.85 | 0.97 | 0.17 | 0.51 | 2.56 | 1.48 | 2.11 | 0.88 | 4.75 | 2.55 |
| LC743491.1 | 0.00 | 0.04 | 0.22 | 0.12 | 0.01 | 0.00 | 0.03 | 0.12 | 0.00 | 0.09 | 0.00 | 0.00 | 0.01 | 0.00 | 0.03 | 0.15 | 0.00 | 0.00 |  | 3.85 | 1.20 | 1.82 | 2.74 | 2.32 | 0.05 | 3.41 | 0.09 | 2.29 | 9.81 | 1.18 |
| LC743492.1 | 0.15 | 0.15 | 0.03 | 0.16 | 0.06 | 0.07 | 0.05 | 0.10 | 0.12 | 1.00 | 0.00 | 0.03 | 0.23 | 0.06 | 1.00 | 0.22 | 0.03 | 0.30 | 0.00 |  | 0.00 | 0.68 | 0.00 | 0.00 | 1.38 | 2.96 | 0.86 | 0.00 | 2.30 | 0.00 |
| LC743623.1 | 1.00 | 1.00 | 0.11 | 1.00 | 0.21 | 0.05 | 0.25 | 1.00 | 0.01 | 1.00 | 0.03 | 0.10 | 1.00 | 1.00 | 0.30 | 1.00 | 0.22 | 0.07 | 0.04 | 1.00 |  | 0.00 | 0.09 | 0.32 | 0.42 | 1.17 | 0.49 | 0.00 | 4.43 | 0.00 |
| LC743839.1 | 1.00 | 1.00 | 1.00 | 1.00 | 1.00 | 0.02 | 1.00 | 1.00 | 0.02 | 1.00 | 1.00 | 0.22 | 0.33 | 1.00 | 0.08 | 1.00 | 0.35 | 0.06 | 0.01 | 0.12 | 1.00 |  | 1.50 | 0.55 | 0.00 | 0.00 | 0.51 | 0.78 | 4.80 | 0.00 |
| LC743840.1 | 0.10 | 0.08 | 0.00 | 0.17 | 0.00 | 0.15 | 0.01 | 1.00 | 0.07 | 1.00 | 0.03 | 0.01 | 0.00 | 0.01 | 1.00 | 1.00 | 0.08 | 0.31 | 0.00 | 1.00 | 0.39 | 0.02 |  | 1.39 | 2.25 | 1.35 | 0.76 | 0.84 | 1.39 | 0.00 |
| LC743841.1 | 0.23 | 0.27 | 0.04 | 0.07 | 0.25 | 1.00 | 0.38 | 0.36 | 0.41 | 1.00 | 0.01 | 0.06 | 0.00 | 0.23 | 0.28 | 1.00 | 0.14 | 0.13 | 0.00 | 1.00 | 0.21 | 0.15 | 0.04 |  | 0.31 | 2.19 | 0.64 | 0.26 | 3.29 | 0.00 |
| LC742716.1 | 1.00 | 1.00 | 1.00 | 0.17 | 1.00 | 0.03 | 1.00 | 1.00 | 0.05 | 1.00 | 0.04 | 1.00 | 0.13 | 1.00 | 0.06 | 1.00 | 0.35 | 0.00 | 0.39 | 0.02 | 0.18 | 1.00 | 0.00 | 0.24 |  | 1.09 | 0.00 | 0.94 | 7.19 | 0.00 |
| LC743471.1 | 1.00 | 0.30 | 0.09 | 0.14 | 0.33 | 0.23 | 1.00 | 0.04 | 0.04 | 0.03 | 1.00 | 1.00 | 0.06 | 0.31 | 0.03 | 0.06 | 0.08 | 0.04 | 0.00 | 0.00 | 0.05 | 1.00 | 0.02 | 0.01 | 0.06 |  | 3.10 | 2.50 | 5.47 | 2.53 |
| LC743472.1 | 0.24 | 0.20 | 1.00 | 0.18 | 0.09 | 0.04 | 0.12 | 0.20 | 0.02 | 1.00 | 0.00 | 0.00 | 0.05 | 0.09 | 0.29 | 0.27 | 0.18 | 0.00 | 0.30 | 0.07 | 0.17 | 0.18 | 0.12 | 0.13 | 1.00 | 0.00 |  | 1.47 | 3.83 | 0.74 |
| LC743473.1 | 0.07 | 0.32 | 0.03 | 1.00 | 0.07 | 1.00 | 0.11 | 1.00 | 0.01 | 1.00 | 0.00 | 0.10 | 0.08 | 0.38 | 0.23 | 0.28 | 0.14 | 0.09 | 0.00 | 1.00 | 1.00 | 0.12 | 0.07 | 0.24 | 0.07 | 0.00 | 0.03 |  | 2.07 | 0.00 |
| LC743493.1 | 0.00 | 0.00 | 0.00 | 0.01 | 0.00 | 0.00 | 0.00 | 0.00 | 0.00 | 0.00 | 0.00 | 0.00 | 0.03 | 0.00 | 0.00 | 0.00 | 0.00 | 0.00 | 0.00 | 0.01 | 0.00 | 0.00 | 0.03 | 0.00 | 0.00 | 0.00 | 0.00 | 0.01 |  | 2.44 |
| LC744194.1 | 0.06 | 1.00 | 1.00 | 1.00 | 0.36 | 0.08 | 1.00 | 1.00 | 0.12 | 1.00 | 0.00 | 0.13 | 1.00 | 1.00 | 1.00 | 1.00 | 0.09 | 0.01 | 0.03 | 1.00 | 1.00 | 1.00 | 1.00 | 1.00 | 1.00 | 0.00 | 0.09 | 1.00 | 0.00 |  |

* *P*-values smaller than 0.05 are considered significant (marked with yellow highlights). An estimate of the disparity index per site is presented for each sequence pair above the diagonal.


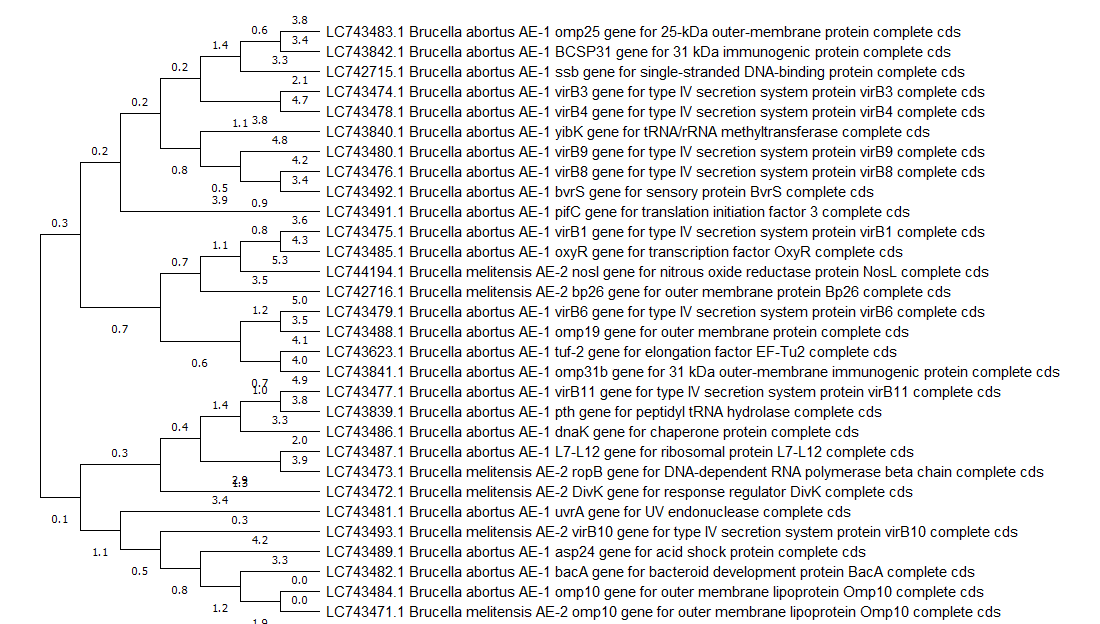


**Fig. S1** The molecular evolutionary estimation was assumed using the Neighbor-Joining method. This research comprised 30 different nucleotide sequences. The total number of sites in the final dataset was 4173.
